# Positron Emission Tomography (PET) in Oncology

**DOI:** 10.3390/cancers6041821

**Published:** 2014-09-29

**Authors:** Andrea Gallamini, Colette Zwarthoed, Anna Borra

**Affiliations:** 1 Department of Research and Medical Innovation, Antoine Lacassagne Cancer Center, Nice University, Nice Cedex 2-06189 Nice, France; 2 Department of Nuclear Medicine, Antoine Lacassagne Cancer Center, Nice University, Nice Cedex 2-06189 Nice, France; E-Mail: maracaz@hotmail.fr; 3 Hematology Department S. Croce Hospital, Via M. Coppino 26, Cuneo 12100, Italy; E-Mail: annaborra@hotmail.it

**Keywords:** FDG-PET, prognosis, oncology

## Abstract

Since its introduction in the early nineties as a promising functional imaging technique in the management of neoplastic disorders, FDG-PET, and subsequently FDG-PET/CT, has become a cornerstone in several oncologic procedures such as tumor staging and restaging, treatment efficacy assessment during or after treatment end and radiotherapy planning. Moreover, the continuous technological progress of image generation and the introduction of sophisticated software to use PET scan as a biomarker paved the way to calculate new prognostic markers such as the metabolic tumor volume (MTV) and the total amount of tumor glycolysis (TLG). FDG-PET/CT proved more sensitive than contrast-enhanced CT scan in staging of several type of lymphoma or in detecting widespread tumor dissemination in several solid cancers, such as breast, lung, colon, ovary and head and neck carcinoma. As a consequence the stage of patients was upgraded, with a change of treatment in 10%–15% of them. One of the most evident advantages of FDG-PET was its ability to detect, very early during treatment, significant changes in glucose metabolism or even complete shutoff of the neoplastic cell metabolism as a surrogate of tumor chemosensitivity assessment. This could enable clinicians to detect much earlier the effectiveness of a given antineoplastic treatment, as compared to the traditional radiological detection of tumor shrinkage, which usually takes time and occurs much later.

## 1. FDG-PET/CT for Tumor Staging

Tumor staging is essential for a modern treatment strategy in oncology. An accurate tumor burden assessment at baseline is required to decide the optimal therapeutic strategy: whether the neoplasm is resectable or, due to a disseminated disease, only palliative treatment could be offered. Second, tumor burden is *per se* a prognostic factor and therefore staging at baseline turns out as a very important prognostic tool. Finally, disease extension definition is essential both for an early (that is, between chemotherapy cycles, often called “interim scanning”) and final tumor response assessment, in the first case during adjuvant chemotherapy to identify ineffective treatment, in the second for final tumor response assessment after an effective treatment. Increasing numbers of patients with newly diagnosed cancer receive primary systemic therapy (so-called neo-adjuvant chemotherapy) followed by surgery. Histopathology provides an accurate assessment of treatment efficacy on the basis of the extent of residual tumor and regressive changes within tumor tissue. However, a variable proportion ranging from 20% to 40% of cancer patients achieve a pathologic complete response, a fact that necessitates methods for monitoring therapeutic effectiveness early during therapy. Interim scanning has an interesting clinical value, generally due to earlier changes in metabolic activity compared to variations in tumor size. Moreover shrinkage usually does not occur immediately after treatment, even in case of chemo-sensitive neoplasms [1]. FDG-PET/CT provides essential information regarding a response to primary chemotherapy: in this setting, baseline FDG-PET/CT is used as reference pre-treatment assessment of tumor extension. Interim FDG-PET/CT is compared to baseline FDG-PET/CT by visual or semi-quantitative assessment by standardized uptake value (SUV) calculation. 

The SUV is calculated in a region of interest (ROI), as of the ratio of the FDG concentration in this area to the injected dose normalized to patient’s body weight [2,3]; it is a relative easy calculation, frequently used in PET reporting and generally accepted as a semi-quantitative index for tumor glucose metabolism. The most widely used parameter is SUV_Max_, defined as the maximal SUV value in the ROI, and reduction in SUV_Max_ (ΔSUV_Max_) has been considered the most reliable indicator of the metabolic activity shutdown. A number of factors are known affecting the SUV calculation such as scanner calibration, clock synchronization between machine and injection time, patient body weight, fasting blood glucose level, image acquisition time, image reconstruction algorithm, partial volume effect, ROI definition [4,5,6]. More recently, new methods have been proposed to assess the burden of metabolically active tumor: the so-called Metabolic Tumor Volume (MTV) and the Total Lesion Glycolysis (TLG). Both parameters have been proposed as reliable indicators of the viable tumor bulk [7]. TLG is the product of median SUV value in a ROI (SUV_Mean_) and MTV; it combines the anatomical and functional information of FDG-PET/CT [8]. The whole neoplastic burden could be assesses by the sum of MTV or TLG of the primary tumor, nodal and distant metastases. To calculate both quantitative parameters a preliminary tumor mapping with a manual contouring of all the tumor lesions by nuclear medicine physicians has been proposed. However, this procedure proved cumbersome when several lesions coalesce in a single bulky tumor mass, and time-consuming for the analytic measurement of SUV_Max_ in every tumor lesion in advanced stage disseminated metastatic disease. New methods based on an adaptive threshold for SUV_Max_ calculation (depending on tumor volume and tumor to background ratio), and semi-automated methods for tumor volume assessment have been proposed [9]. 

### 1.1. Lung Cancer

Staging Non-Small Cell Lung Cancer (NSCLC) by FDG-PET/CT is probably one of the main daily-practice indications encountered in a nuclear medicine department. Indeed, several non-invasive imaging modalities are available for staging NSCLC, but FDG-PET/CT utility and advantages have been clearly demonstrated long since. The addition of FDG-PET/CT to the conventional staging assessment was reportedly shown to change the management in 20%–30% of patients with NSCLC, mostly by upstaging disease and, notably, by redefining unresectable a previously defined resectable disease by traditional radiological means [10,11]. Several recent studies [12], and in particular the randomized multicenter study of Maziak *et al.* [13], reported that tumor staging with FDG-PET/CT immediately before surgery revealed more patients with mediastinal and distant metastatic disease than conventional imaging; disease was correctly upstaged in 23 of 167 FDG-PET/CT and in 11 of 162 conventional staging imaging modalities. Likewise, Fischer *et al.* noticed that the use of FDG-PET/CT for preoperative staging of NSCLC reduced both the total number of thoracotomies and the number of futile thoracotomies but did not affect overall mortality [14]. 

#### 1.1.1. T Staging

FDG-ET/CT provides information on tumor staging according to TNM criteria. The utility of FDG-PET/CT for determining T stage, and in particular T3 or T4 invasion, has not been definitely determined. The evaluation of tumor spread to the pleura by FDG-PET/CT is probably the main advantage compared to conventional imaging. Actually, pleural effusion is relatively frequent in patients with NSCLC, and may be malignant or benign, in particular in patients with post-obstructive pneumonia. The sensitivity and specificity of FDG-PET/CT in determining pleural invasion range from 70% to 95% and 64% to 94% respectively [15]. The limitations of FDG-PET/CT for T staging are due to the anatomical localization and size measurement difficulties, microscopic disease underestimation, or absence of FDG uptake in case of low-metabolism tumors (bronchoalveolar cell carcinoma, carcinoid tumors). Nevertheless, FDG-PET/CT turned out the most accurate tool for T staging assessment, with a correct T staging definition in 86% of patients, as compared to 68% with computed tomography (CT) alone [16].

#### 1.1.2. N Staging

Functional imaging with FDG-PET/CT proved to be superior to contrast-enhanced CT (CeCT) for N staging, in particular by adding metabolic information able to disclose morphologically undetectable nodal dissemination, ultimately increasing specificity and positive predictive value of N staging [17]. For example, in a prospective study (106 patients with NSCLC), the sensitivity, specificity and accuracy was higher with FDG-PET/CT (respectively 85%, 84% and 84%) than with CeCT alone (respectively 70%, 69% and 69%) [18]. However, the sensitivity of N staging by FDG-PET/CT remains disappointingly low (45%) and false negative cases have been reported [19], particularly for lymph nodes size <10 mm (Se = 32.4%) compared with lymph nodes >10 mm (Se = 85.3%). Other limitations are the false-positive rates due to unspecific FDG uptake like inflammation or granulomatous disease (e.g., sarcoidosis), leading to a reduction in specificity [15]. Despite the above improvement in accuracy of N staging with FDG-PET/CT, surgical staging remains the standard, especially to detect occult mediastinal nodal invasion [20,21]. For these reasons, both endobronchial ultrasound-guided transbronchial needle aspiration (EBUS-TBNA) and endoscopic ultrasound-guided fine-needle aspiration (EUS-FNA) have been recommended as essential tools for tumor staging after FDG-PET/CT [22].

#### 1.1.3. M Staging

Long since, FDG-PET/CT proved very informative on metastatic spread in NSCLC, able to detect unsuspected distant metastases in up to 28% of patients [23], and to impact in a relevant way the treatment plan in as many as 53% of cases [24]. FDG-PET/CT is for example useful for differentiating benign from malignant adrenal lesions, with a sensitivity and a specificity reported by Erasmus *et al.* of 100% and 80%–100% respectively [25], though in some cases a second imaging technique was needed [26]. FDG-PET/CT is also accurate for detecting bone metastasis, with an even higher accuracy than Magnetic Resonance Imaging (MRI) and bone scintigraphy (BS) in some publications [27]. 

### 1.2. Colorectal Cancer (CRC)

FDG-PET/CT is not routinely used in the staging of colorectal cancer, but could be proposed for problem solving, or in the presence of CEA elevation or resectable metastases [28], as proposed in the National Comprehensive Cancer Network guidelines (NCCN version 4.2013 [29]). Actually, for the local T staging, MRI and endorectal ultrasound (ERUS) are recommended [30]; for the detection of colorectal metastases (N and M staging), the most frequently used modalities are US, CT, MRI and FDG-PET/CT [31]. 

In spite of the well-known limits of these metrics, ERUS, CT and MRI use a purely dimensional criterion to detect a nodal involvement [30]. However both criteria, functional and dimensional are still needed for N staging: FDG-PET/CT may provide additional metabolic information, but has limits essentially due to its spatial resolution, giving a lack of sensitivity. For example, in a Japanese study including 88 patients, FDG-PET/CT improved accuracy of preoperative lymph node involvement detection compared to nodal diameter, with a sensitivity and specificity of 51% and 85% for local lymph nodes and 62% and 92% for distant lymph nodes [32]. 

FDG-PET/CT seems to have only a limited value for M staging in CRC: for example, a meta-analysis of prospective studies (3,391 patients) aimed at assessing the role of imaging to detect liver metastases [33], and showed that MRI imaging is the preferred choice modality in patients who have not previously undergone therapy, in particular in evaluating lesions less than 1 cm (sensitivity 80%–88% and specificity 93%–97%). FDG-PET/CT can be used as the second-line modality (sensitivity 81%–94%, higher than CT), but data about this modality were too limited for comparisons with others.

FDG-PET/CT is not used routinely to detect lung metastatic spread of CRC; it may be accurate, especially when nodules have a sufficient size (>9 mm), with a sensitivity and specificity reported by Bamba *et al.* of 57.1% and 99.1% [34].

In patients with potentially operable metastatic colorectal cancer, FDG-PET/CT has a valuable role by improving staging accuracy and characterizing indeterminate lesions; in the study of Briggs *et al.*, it could have a major impact on subsequent management in 30% of patients, and a minor impact in 12% of patients. Following FDG-PET/CT, as many as 35% of patients were no longer considered for surgery [35]. Likewise, another study of 341 patients with potentially resectable liver and/or pulmonary CRC metastases observed that FDG-PET/CT upstaged disease in 33.1% and down staged disease in 24.9% compared to conventional imaging. As a consequence, surgery was averted in 33.8% patients, and FDG-PET/CT showed an overall sensitivity of 87.1% and specificity of 88% in detecting metastatic disease [36], in line with other observations [37,38].

A systematic review and economic evaluation recently concluded that there is insufficient evidence to authorize the use FDG-PET/CT routinely in primary CRC, but that using this imaging modality as a complementary imaging technique is cost-effective in the pre-operative staging of recurrent CRC (pooled sensitivity of 91% and specificity of 91%) and in case of suspected metastatic disease (pooled sensitivity of 91% and specificity of 76%). Although FDG-PET/CT may change patient management in some cases, we have to keep in mind that, at this time, the data are still discordant and the quality of studies is generally poor [39].

### 1.3. Esophageal Cancer

The role of FDG-PET/CT in esophageal cancer staging is still unsettled. According to the highlights of the EORTC St. Gallen International Expert Consensus on the primary therapy of gastric, gastroesophageal and esophageal squamous cell cancer (SCC) [40], there was uncertainty about the role of FDG-PET/CT as part of routine preoperative staging. Its value may rely in the detection of otherwise undiscovered distant metastases which could contraindicate the surgical approach, and in facilitating treatment planning for radiotherapy, but it is unclear if FDG-PET/CT scans add to the accuracy of state-of-the-art high quality CT to detect locally invasive tumour bulk. For the adenocarcinoma of the gastroesophageal junction and the esophagus, the prevailing recommendation is to stage patients with gold standard techniques such as endoscopy, CT, EUS and FDG-PET/CT.

For the local tumor extent, endoscopy, EUS, CT scan and MRI are classically recommended [41], and FDG-PET/CT alone is not routinely recommended. Both adenocarcinomas and SCCs have high FDG avidity, but false positive uptakes may be caused by esophagitis or post-dilatation, and false negative results may be encountered in small tumors [42]. EUS remains the imaging modality of choice for T staging because of its superior resolution.

For N staging, integrated FDG-PET/CT may improve the positive predictive value (PPV) of regional lymph nodes staging when compared with CeCT (PPV = 93.8% for FDG-PET/CT, *versus* 62.5%–73.7% for CeCT) [43]. Vazquez Sequeiros suggested that EUS is the most accurate technique for preoperative local-regional staging of esophageal carcinoma, once the CT and/or the PET have excluded the presence of distant metastasis [44]. According to this review, overall accuracy for N staging was 69% for CT, 56% for FDG-PET/CT, and 81% for EUS. EUS was the most sensitive technique, whereas CT and FDG-PET/CT were more specific tests. A more recent meta-analysis confirmed the limited accuracy of FDG-PET/CT for N staging, with a pooled sensitivity and specificity of 62% and 96% [45]. Another recent work underlined that the variable FDG avidity of the primary esophageal tumor could affect the detectability of lymph nodal metastases in esophageal cancer with a low metabolic activity [46]. FDG-PET/CT has a higher accuracy than CeCT for M staging; a meta-analysis observed that FDG-PET/CT has a sensitivity of 71% and specificity of 93% in the detection of distant metastases, in comparison to 52% and 91% for CT respectively [47]. When tumor spread was assessed with FDG-PET/CT using quantitative parameters such as SUV_Max_, significant variation in TNM staging was evident especially for nodal lesions [48]. In a recent prospective study of 139 consecutive patients (all stages), FDG-PET/CT changed the stage group in 56 of 139 (40%) patients and changed management in 47 of 139 (34%) patients. FDG-PET/CT has also prognostic stratification in the primary staging of esophageal cancer [49].

Finally, baseline FDG-PET/CT was shown in preliminary reports, to have an independent prognostic value on esophageal cancer treatment outcome when PET-derived quantitative parameters were used for prognostic patient stratification. The latter included SUV_Max_ and survival outcomes [50], FDG-PET/CT N stage [51] or pretreatment MTV and overall survival [52].

### 1.4. Gastric and Gastroesophagal Cancer

According to the highlights of the EORTC St. Gallen International Expert Consensus, the panellists agreed that there is currently no indication for FDG-PET/CT scans in routine staging of gastric cancer [40]. For preoperative T staging, EUS remains the choice modality, while both CT and PET are most useful to evaluate distant metastases [53], with variability sensitivity ranging from 33% to 81% and 47% to 81% respectively, and specificity ranging from 82% to 96% and 89% to 91% respectively. However, notably, FDG avidity was shown to depend on tumor histologic subtype; FDG-PET/CT has a significant lower sensitivity for diffuse type histology (mucinous, signet ring) than for the intestinal (non-mucinous) tumors [54]. In a similar way to some lymphoma subset, this observation may limit the value of FDG-PET/CT for staging in those cases. Another limitation of FDG-PET/CT staging is the false negative cases due to occult peritoneal dissemination. The rate of occult peritoneal disease varies from 20% to 25% in the literature [55], and laparoscopy and diagnostic washings prior to surgery may be warranted for patients who demonstrate advanced pathology at diagnosis (T3 or more, N+) without evidence of metastatic dissemination.

### 1.5. Pancreatic Cancer

Multimodality imaging is critical in the diagnosis and management of pancreatic cancer (PC). FDG-PET/CT is increasingly viewed as a useful and accurate modality in diagnosing, staging and managing this neoplasm, but further studies are warranted at this time to confirm that [56]. 

For diagnosing PC, FDG-PET/CT has an acceptable pooled sensitivity and specificity of 91% and 81% respectively, according to a recent meta-analysis (30 studies, 1582 patients) [57]. The SUV_Max_ of FDG-PET/CT can be used in the differential diagnosis of solitary pancreatic lesions and can also help in the prediction of proliferative activity of pancreatic cancer [58]. Indeed, in several observations, higher SUV_Max_ of primary pancreatic tumor is associated with poor prognosis [59,60]; metabolic tumour burden like MTV and TLG may be some prognosis factors too [61]. As regards histologic subtypes, a recent study suggests that not only pancreatic ductal adenocarcinoma, but also solid pseudopapillary tumor has an increased FDG metabolism [62]. Like in other tumors, PC should sometimes be distinguished from inflammatory lesions mimicking cancer such as mass-forming pancreatitis: in this settings FDG-PET/CT proved of limited value either because the SUV_Max_ values of the inflammatory lesion overlapped with those detected in pancreatic cancer [63], and the small volume of both lesions [64]. FDG-PET/MRI fusion image may significantly improve accuracy compared with that of FDG-PET/CT (96.6% *vs.* 86.6%) [65]. For evaluation of intraductal papillary mucinous neoplasms (IPMN), FDG-PET/CT seems to be promising in distinguishing benign from malignant lesions, and therefore for selecting patients for surgical treatment or for long-term follow-up [66,67]. For N and T staging of the disease, the accuracy of FDG-PET/CT remains unclear. According to the meta-analysis of Wang *et al.*, the sensitivity and specificity for N stating are 64% and 81% respectively (four studies, 101 patients), and for liver metastasis 67% and 96% (seven studies, 316 patients) [57]. A number of reports focused on the role of FDG-PET/CT for early treatment response assessment, with interesting results for staging the disease [68]. For example, Topkan *et al*. observed that FDG-PET/CT alters initial management decisions in 36.6% of patients with locally advanced pancreatic carcinoma planned to undergo chemoradiation; in 26.8% of patients, FDG-PET/CT restaging showed distant metastases not detected by conventional staging [69].

### 1.6. Head and Neck Squamous Cell Carcinoma

The preoperative staging of squamous cell head and neck cancer (HNSCC) includes clinical examination and imaging methods like CeCT or MRI. Even these techniques could detect morphological invasion, small tumors are far better detected with FDG-PET/CT [70]. We should notice that necrotic lesions do not accumulate FDG, and diagnostic CeCT may be helpful for correct local staging in that case [71]. For N staging, a N plus stage cannot be ruled out by FDG-PET/CT, even with multiple time point dynamic imaging techniques, and surgeons should continue to rely on clinical evaluation to stage the patients and consider surgical staging if nodal metastases are clinically suspected [72]. Nevertheless, the clinical impact of FDG-PET/CT used for head and neck initial staging has been demonstrated in several publications. For example, in a prospective investigation (76 patients), FDG-PET/CT led to a TNM classification alteration in 34%, and a change in radiotherapy planning technique and/or dose in 29% [73]. Another observation related that the accuracy of PET and PET/CT for detecting primary tumors and cervical metastases was comparable, but significantly higher than that of CT/MRI (98%–97% *vs.* 86%–88% for primary; 92%–93% *vs.* 85%–86% for neck) [74]. Several other studies also demonstrate that pretreatment FDG-PET/CT is superior to conventional imaging and could alter the TNM stage in about 30% [70]. Likewise, in patients with suspected recurrent head and neck cancer, a recent meta-analysis showed that FDG-PET/CT has high sensitivity (92%), specificity (95%) and accuracy (97%) for screening distant metastasis before salvage treatment [75]. In addition to that, FDG-PET/CT may also detect other primary tumors like lung, gastric and esophageal cancer [76]. To summarize, FDG-PET/CT is a useful technique in HNSCC for planning the most appropriate treatment, offering the possibility to detect the primary tumor, locoregional and distant metastatic involvement, as well as another primary malignancy.

### 1.7. Breast Cancer

The role of FDG-PET/CT in tumor staging and restaging is steadily increasing along with the body of evidence regarding its impact in breast cancer management [77]. The ability of FDG-PET/CT to detect primary tumor, locoregional and distant metastasis is described in current literature.

The initial breast cancer evaluation is commonly made by mammography, US and MRI. Even if multiple studies have shown the high accuracy of FDG-PET/CT in detecting suspected primary breast malignancy [78,79], we should remember that it is exclusively reserved as a staging tool for cases of pthology-proven disease [77]. Indeed, false negative cases have been reported, in case of tumor-size <20 mm, or lobular carcinoma subtype [79]. False positive cases could also be found, like fibroadenoma, inflammatory processes, gynecomastia, mastitis, granulomas, post-surgical changes, radiation necrosis or lactation [80]. Regarding the primary tumor, quantitative analysis has been correlated with histopathology characteristics. A higher SUV_Max_ is correlated with more frequently detected distant metastases, aggressive histologic architecture, triple-negative receptor phenotype [81]. Groheux *et al.* also described the relationship between SUV_Max_ and histologic grade (median of 9.7 for grade III) [82], and other studies noticed the more aggressive and prognostically poor invasive ductal carcinomas have increased average SUV_Max_ relative to invasive lobular carcinomas [77]. 

For N staging, the imaging-guided sentinel lymph node biopsy (SLNB) with Tc^99^colloid remains the gold standard, with high sensitivity and specificity for nodal disease detection. In patients with early-stage disease and clinically negative axillary nodes, the SLNB is recommended [83]. However, the search for a non-invasive technique has led to several studies evaluating the efficacy of conventional and/or functional imaging. The sensitivity of FDG-PET/CT in this application is variable in the literature. A meta-analysis (862 patients) observed a mean sensitivity of FDG-PET/CT of 56% and a mean specificity of 96% [84]; PET performed less well for small metastases (mean sensitivity: 11% for micro metastases ≤2 mm, and 57% for macro metastases >2 mm). In this study, MRI showed a higher sensitivity in detecting occult metastases than FDG-PET/CT. In another study (311 patients), a better sensitivity was observed (sensitivity = 82%, specificity = 92%) in evaluating axillary nodes [85]. Although the limited sensitivity of FDG-PET/CT for nodal staging, it generally outperforms conventional imaging regarding the detection of extra-axillary lymph node metastases [86]. Moreover, the specificity and positive predictive value of FDG-PET/CT are high (often exceeding 90%) [77], which means that, in case of an FDG-avid axillary node, the sentinel lymph node procedure could be skipped and an immediate axillary lymph node dissection can be planned [87]. FDG-PET/CT may also detect other nodal metastases sites, like internal mammary chain and periclavicular chain [85]. It could therefore play a role in nodal staging of patients with clinical and/or histological risk factors. In pretreatment evaluation of breast cancer, several studies observed that FDG-PET/CT may change the initial staging compared to conventional imaging. For example, in a prospective study (106 patients with primary tumors larger than 3 cm), FDG-PET/CT changed the initial staging in 42% of patients, and in 14% of patients, sites of extra-axillary malignancy were only detected by FDG-PET/CT. A treatment modification based on FDG-PET/CT results was done in 8% [79]. Other publications underlined that FDG-PET/CT findings could have a clinical impact in breast cancer, like Riegger *et al.*, who found that 14% of patients had a change in the disease management according to PET results [88]. In M staging, bone scintigraphy (BS) has traditionally been used as the first-line imaging technique for bone involvement despite its limited sensitivity for detecting pure lytic metastases [89]. Many studies suggest the superiority of FDG-PET/CT *versus* conventional BS, and the superiority or equivalence of FDG-PET/CT *versus* MRI imaging [77]. For example, in a study examining 132 bone lesions, the sensitivity of FDG-PET/CT was higher than BS (respectively 96% and 76%) [90]. FDG-PET/CT proved very useful for lung metastases detection, with the limits of a very low sensitivity for nodules with a largest diameter ≤8 mm [77], for visceral metastases, like adrenal masses with the exception of benign FDG-avid solid masses [91], or for liver metastasis, albeit MRI proved superior (like in colorectal cancer) [92]. Despite its high accuracy in breast cancer staging, FDG-PET/CT failed to prove cost-effective in baseline staging in the asymptomatic patient where other imaging modalities proved superior and could not be replaced by PET [77]. However, the benefits of FDG-PET/CT in the initial evaluation of breast cancer should be considered, and the choice of the staging imaging strategy should be done taking into account the clinical/histological risk factors.

### 1.8. Other Gynecological Malignancies

#### 1.8.1. Ovarian Cancer

There is mounting evidence that FDG-PET/CT has an increasing role in the management of ovarian cancer, with its main indication to detect tumor recurrence in presence of rising CA-125 serum values and negative conventional imaging studies [93]. The benefits of the use of FDG-PET/CT in these settings has been reported several times in the literature [94,95], with a sensitivity of more than 90% in detecting occult metastases. In the study of Zimny *et al.*, FDG-PET/CT preceded the conventional diagnosis by a median of 6 months in patients judged clinically free of disease. Menzel *et al.* suggest that a PET indication is worthwhile at CA 125 levels of approximately 30 U/mL [96]. A more recent prospective multi-center, cohort study (90 patients) confirmed the impact of FDG-PET/CT in suspected recurrent ovarian cancer, which affected disease management decisions in 60% of the cases (in 49% with a high, in 11% with a medium clinical impact) with a much higher detection rate compared to conventional imaging [97]. 

For the characterization of asymptomatic adnexal findings, FDG-PET/CT has no place due to lack of sensitivity [98], and MRI remains the best imaging modality choice.

For the initial staging of ovarian cancer, FDG-PET/CT is not routinely used. Nevertheless, some publications noticed that it could be interesting in advanced epithelial ovarian cancer, in particular for the detection of supradiaphragmatic lymph node metastases like parasternal lymph nodes, with better accuracy than conventional CT (detection rate: 67% *vs.* 33%) [99]. However, increased mediastinal FDG uptake was not shown to play a significant prognostic role, while complete cytoreduction did [100]. For the initial preoperative staging of ovarian cancer, FDG-PET/CT may be superior compared to CT alone [101,102], but some publications also observed limits, as De Iaco *et al.*, who reported a sensitivity and specificity of 78 and 68% respectively, with a high rate of false negative results in lesions <5 mm such as found in presence of peritoneal carcinomatosis [103]. 

However, conflicting results have been reported on the sensitivity of FDG-PET/CT scan in detecting peritoneal carcinomatosis; Turlakow, Suzuki and Kim reported higher diagnostic accuracy of FDG-PET/CT than CeCT in this settings, with a sensitivity and specificity for FDG-PET/CT of 67%–92.2% and 90%–94% respectively, as compared to 22%–88.5% and 65%–77% respectively for CeCT [104,105,106]. The sensitivity of FDG-PET/CT proved also similar to that of conventional MRI, and even better for detecting small peritoneal lesions (<2 cm) in patients with recurrent ovarian cancer [107]. However, FDG-PET/CT has limits, in particular for the detection of small peritoneal implants (<5 mm) because of the limited PET resolution, and surgical staging remains the gold standard [108]. The good performances of FDG-PET/CT in detecting peritoneal carcinomatosis lead to interesting information for optimizing patient selection for cytoreductive surgery in recurrent ovarian cancer; recently, Ebina *et al.* observed that FDG-PET/CT led to a change in management plan in 58.4% in that case, with a total number of patients in whom cytoreductive surgery was selected as the treatment of choice increased from 12 to 35 according to FDG-PET-CT results [109]. In the preoperative management, FDG-PET/CT is also able to detect distant metastases (25/95 patients upstaged from FIGO stage III to stage IV by FDG-PET/CT in a recent study [110]. However, upward stage migration did not worsen the prognosis of stage III patients, and in advanced ovarian cancer, the only prognostic factor that retained a significant prognostic value is the quality of response to cytoreductive therapy. Another study proposed FDG-PET/CT criteria such as FDG-PET/CT stage IV, pleural exudates, and PET-positive large bowel mesentery implants, which were statistically significant in the prognosis univariate analysis to guide the administration of neo-adjuvant chemotherapy in advanced ovarian cancer, but, once again, incomplete tumor debulking was the only statistically significant independent prognostic variable using multivariate analysis (*p* = 0.0001) [111]. Other prognostic factors like MTV or TGL may be interesting, but more data are needed at this time to confirm that [112].

#### 1.8.2. Uterine Cervical Cancer

FDG-PET/CT has an increasing role in the primary evaluation of uterine cervical carcinoma, in particular for evaluating lymph node status and distant metastases [113]. 

The positive diagnosis of cervical carcinoma is established by clinical examination and cervical biopsy. The local extent of the disease is usually assessed by colposcopy, while parametrical and soft tissue invasion is detected by MRI, which has a high soft-tissue contrast resolution.

Cervical carcinoma is usually highly FDG avid, and the primary tumor may be observed on FDG-PET/CT, but its value is limited in defining local extent compared to MRI. On the other hand, FDG-PET/CT can be used for the initial evaluation of lymph node involvement [114], in particular in advanced stage disease. Indeed, FDG-PET/CT proved more accurate than CT alone for N staging, depending on the tumor stage [115], with sensitivity and specificity values of 53%–73% and 90%–97% respectively in early stage [116,117], and of 75% and 95%, respectively in advanced stage [118]. 

A major issue in N staging is the relative weak sensitivity of anatomical and functional imaging for tumor detection, especially in case of microscopic nodal invasion, with a number of false negative results with FDG-PET/CT as high as 22% in case of para-aortic and pelvic occult metastases [119]. On the other hand, standard-technique MRI imaging showed a lower sensitivity than FDG-PET/CT (30.3% *vs.* 57.6%) [120], while Diffusion Weighted MRI (DW-MRI) proved superior (83.3% *vs.* 38.9%) [121]. Consequently, surgical staging is not necessary in presence of a positive FDG-PET/CT, while it remains the gold-standard procedure in case of a negative scan. Stage migration during cervical cancer staging occurs mainly for occult distant nodal invasion: nodal involvement at the highest level in para-aortic, retrocrural, supraclavicular areas is a significant prognostic factor [122]. The pre-operative tumor metabolic activity assessment by SUV_Max_ measurement seems to have a prognostic role in uterine cervical cancer [114,115,116,117,118,119,120,121,122,123]. 

Despite significant improvement in tumor detection, FDG-PET/CT failed to supersede surgical staging in baseline tumor extension assessment. Gouy *et al.* [124] assessed the role of extended-field radiation on para-aortic nodes combined with chemotherapy in PET-negative patients, showing that disease outcome was similar to that of patients without extra-pelvic nodal invasion. These data seem to warrant the utility of surgical staging in PET-negative patients, albeit tumor spread in para-aortic nodes is rare in presence of PET negative scan (8% *vs.* 18% in PET-positive patients) [125]. 

FDG-PET/CT proved superior to conventional radiological techniques for M staging, like for example in case of bone metastases [126], leading to a modification in radiotherapy dose and fields in 34% and of the overall therapeutic strategy in 23% of the patients [127].

#### 1.8.3. Melanoma

Several studies witnessed over the last decade the utility of FDG-PET/CT in the management of patients with advanced melanoma [128,129]. For example, in a study of 38 patients with melanoma stage II or III, a sensitivity of 97% and a specificity of 56% were calculated respectively for FDG-PET/CT, compared with 62% and 22%, respectively for other imaging modalities, resulting in stage migration in 34% of the cases [130], suggesting that FDG-PET/CT could replace the standard battery of imaging tests currently performed on high risk melanoma patients [131,132].

#### 1.8.4. Lymphoma 

The above-mentioned considerations on physiopathology of tracer uptake and mechanism of image generation in PET scan are the underpinnings of the functional imaging concept. Moreover, they could explain the higher accuracy of FDG-PET/CT in baseline lymphoma staging compared with traditional anatomical imaging techniques, such as CT or MRI. Fused FDG-PET/CT is by far the more accurate imaging strategy compared with CeCT or PET alone [133,134]. By upstaging one of three or four patients with lymphoma, FDG-PET/CT could, in theory, significantly improve final outcomes [135]. Most of the existing evidence in the literature on the contribution of FDG-PET/CT in lymphoma staging comes from studies performed in Hodgkin lymphoma (HL). FDG-PET/CT proved to be a more sensitive and specific imaging technique than other conventional modalities, including Gallium and CT for determination of extent of nodal and extranodal disease [136]. Stage migration occurs in nearly 25% of patients, mostly to upstage disease, leading to a change in treatment strategy in nearly 10%–15% of them [137]. The role of CeCT performed simultaneously in the same diagnostic session with the PET scan (PET/CeCT) is still a subject of debate. Direct comparison of unenhanced, low-dose, FDG-PET/CT and PET/CeCT did not show statistically significant differences in the number of detected nodal and extranodal sites, but lymphoma was occasionally upstaged with the help of CeCT or additional clinically relevant findings were identified [138,139]. PET/CeCT may be useful in patients with abdominal and pelvic involvement for delineating lymph nodes from adjacent bowel loops and vasculature [140].

As far as the contribution of functional imaging in Diffuse Large B-Cell lymphoma (DLBCL) staging, the overall accuracy of CeCT and FDG-PET/CT in lymphoma staging was assessed in a head-to-head comparison of both techniques by Elstrom *et al.* in a mixed series of 37 HL and 38 DLBCL patients. FDG-PET/CT detected additional lesions in one third of the patients staged with CT, and in 33% an increased clinical stage was demonstrated. Lymphoma therapy changed based on FDG-PET/CT in two patients. In contrast, diagnostic CT detected few splenic lesions, without any stage migration [141].

In the PET era, the role of bone marrow trephine biopsy (BMB) for lymphoma staging has been questioned. In a recent retrospective study, the role of routine BMB was assessed in a cohort of 454 HL patients staged with FDG-PET/CT: BMB upstaged only five patients from stage III to IV and no BMB allocated patients in another treatment or risk group [142]. Although BMB remains essential for the diagnostic workup, bone marrow involvement is a rare finding at disease onset in HL, thus in patients staged with FDG-PET/CT, BMB should no longer be a routine procedure.

As for HL disease, FDG-PET/CT is being routinely used in DLBCL staging for its ability to evaluate nodal and extranodal disease sites like skeletal, liver and lung involvement. Recently, in a retrospective review conducted on 130 DLBCL patients, Khan *et al.* found a higher overall accuracy of FDG-PET/CT in detecting bone and bone marrow (B/BM) invasion by lymphoma compared with BMB, with a sensitivity and specificity of 94%, 100%, and 40%, 100%, respectively. The negative and positive predicting value was 98% and 100%, respectively. However, these data should be interpreted with caution. In fact, the authors claimed that the criterion for bone marrow involvement was either histologically-proven DLBCL presence in the marrow biopsy or PET-positive B/BM involvement with a focal, focal and diffuse, or diffuse-only pattern of FDG uptake, irrespective of iliac crest biopsy or the pattern diffuse or focal of FDG uptake [143]. These results were somehow criticized or attenuated by Avigdor *et al.* who pointed out that bone marrow involvement detected by FDG-PET/CT has a similar Progression-Free Survival (PFS) and Overall Survival (OS) to those individuals with stage IV disease without an involved marrow, whereas marrow involvement identified by BMB was associated with a worse outcome [144]. In conclusion, although BMB involvement is largely predicted by FDG-PET/CT results, BMB remains essential for the diagnostic workup in DLBCL, especially in cases of discordant lymphoma where the low grade component of BM invasion could hardly be detected by FDG-PET/CT. Prognostic value of FDG-PET/CT in baseline staging also has been reported in Follicular Lymphoma (FL, grade 3 more specifically). In a mixed clinical-imaging prognostic score containing (1) osteo-medullar uptake; (2) SUV_Max_ ≥ 15; (3) extranodal involvement and (4) largest diameter of lesion ≥ 7 cm, number of nodal sites affected on PET ≥6, Le Dortz *et al.* found that a score value ≥ 2, whatever the Follicular International Prognostic Index (FLIPI) score, was the only predictive factor in multivariate analysis on treatment outcome. Furthermore, FDG-PET/CT detected more lesion than CT especially for lymph node involvement (+51%) and extranodal lesions (+89%) resulting in patient upstage in only 11% of the cases [145]. In the Italian prospective study FOLL-05, upward stage migration upon PET scan occurred in up to 62% of limited-stage FL patients, who otherwise would have been treated with radiation therapy alone, resulting in a treatment change in a relevant portion of the patients [146]. For Mantle Cell Lymphoma (MCL), the combination of SUV_Max_ and MCL-adapted international prognostic index (M-IPI) was able to stratify patients into three prognostic groups: low (29%; no relapse/progression), intermediate (42%; median Event-Free Survival (EFS): 37 months), and high risk (29%, median EFS: 22 months) of treatment failure (*p* = 0.004) [147]. High FDG-uptake was related to the presence of blastoid and large cellular variants of MCL, which are known to have a more aggressive disease course compared with common MCL [148]. Conversely, the low sensitivity of FDG-PET/CT in detecting bone marrow and/or gastrointestinal involvement by lymphoma did not lead to significant upward stage shift. In conclusion, FDG-PET/CT is strongly recommended before treatment onset for patients in routine staging workup in most lymphoma subset with the exception of marginal extranodal lymphoma, cutaneous lymphoma, and small lymphocytic/Chronic Lymphocytic Leukemia (SLL/CLL) lymphoma [149] (See Table 1). 

**Table 1:** FDG-PET/CT for solid tumor staging.

Tumor Type	Diagnosis	Staging	Prognosis
Lung	++ (solitary lung nodule) − (bronchoalveolar cell carcinoma, carcinoid tumor = low FDG avidity)	T: +/− N: + (EBUS/TBNA, EUS/FNA or histology often required) M: ++	+ (SUV_Max_, MTV)
Breast	+/− (routinely not used)	N: +/− (SLNB = gold standard for early stage) M: ++ (if advanced stage or risk factors)	+ (SUV_Max_: correlation with histopathology, distant metastasis)
Ovarian cancer	− (lack of sensitivity)	Initial: − routinely not used, discussed in advanced disease/suspicion of peritoneal carcinomatosis Recurrence: ++ (CA125 elevation)	+ (?), more data needed (MTV, SUV_Max_)
Cervical carcinoma	−	T: − (MRI better) N: ++ in advanced disease (but surgical staging generally required if PET negative) M: ++ (advanced stage)	+ (SUV_Max_, lymph node involvement localization)
HNSCC	++ (cervical lymph node of unknown primary tumor)	T: + N: + (if PET negative, surgical staging generally requires) M: ++	+ (more in treatment response evaluation)
Colorectal cancer	−	− (routinely not used) + discussed if potentially operable metastasis, problem solving or CEA elevation (recurrence)	+ (?) more data needed
Esophageal cancer	−	squamous cell cancer: +/− adenocarcinoma: +	+
Gastric cancer	− (lower FDG avidity for diffuse type histology (mucinous, signet ring))	−	More data needed
Pancreatic cancer	+ (differentiating benign/malignant, IPMN)	+/− (not routinely used, might be discussed)	+ (SUV_Max_, MTV, TGL)
Melanoma	−	++ (advanced stage) ++ (surveillance if advanced stage)	+

Legend of Table 1: −: PET not routinely recommended; +: for staging, PET indication may be discussed; interesting for prognosis; ++: PET recommended.

## 2. FDG-PET/CT in Solitary Pulmonary Nodule (SPN)

Solitary Pulmonary Nodule (SPN) is one of the most frequent incidental findings (14.8% of asymptomatic patients) [150], and could have both a benign or malignant origin. CeCT of high-resolution CT scan (HRCT) may give information on the morphologic characteristics of SPN (size, border, calcification, intra-nodular fat) and its size change, but this imaging modality has limits; 25%–39% of malignant nodules are inaccurately classified as benign [151,152]. The gold standard for diagnosing SPN is pathology, with a tissue sample obtained either surgically or by biopsy. 

FDG-PET/CT is a non-invasive diagnostic tool which gives metabolic evaluation of the SPN, and may reduce the numbers of unnecessary samples. 

A qualitative and quantitative (SUV measurement) assessment of the SPN metabolic activity can be interpreted on FDG-PET/CT. The SUV_Max_ was shown to be predictor of the neoplastic nature of the tissue: in a large (585 patients) prospective study, 496 patients with a median SUV_Max_ of 8.5 (range, 0 to 36) had a malignant neoplasm, and 89 patients with a median SUV_Max_ of 4.9 (range, 0 to 28) had a benign lesion (*p* < 0.001). False negative FDG-PET/CT findings were: broncho-alveolar carcinoma (11), carcinoid (4), and renal cell (2). False positives findings were related to fungal infections (16) [153]. The threshold of SUV_Max_ for distinguishing benign from malignant lesions is variable among the literature, and therefore lacks reproducibility. For example, Lowe *et al.* observed good performances of FDG-PET/CT with the most frequently SUV_Max_ threshold used in the literature, which is 2.5 (overall sensitivity and specificity for detection of malignant nodules of 92% and 90%) [154]. 

A same cut-off value has been used in other publications, like Hashimoto *et al.*, who calculated a sensitivity of 100%, specificity of 63%, positive and negative predictive values of 62% and 100%, respectively. When an SUV of 1.59 was the cutoff for positive FDG-PET/CT results, the ROC analysis revealed a lower sensitivity (81%), but higher specificity (85%) [155]. In this study, the probability of malignancy in any visually evident lesion was about 60%. Other studies suggest a higher cut-off value (SUV_Max_ > 3.5) [156], or a visual interpretation by experienced physician [157]. This variability among the different studies is probably explained by the fact that SUV may be affected by a large number of parameters, like equipment used, physic and biological factors. In most studies, the sensitivity of FDG-PET/CT is higher than its specificity [150]; FDG is a marker of glucose metabolism, and is not specific of neoplastic disease. Many benign abnormalities can produce false-positive findings on PET/CT, like granulomatosis, infection or inflammation. Consequently, in endemic regions of infectious or granulomatous lung diseases, FDG-PET/CT has significant limits. To give an example, a study of 279 patients in south-central United States with high prevalence of histoplasmosis, the specificity of PET/CT was only of 40% [158]. Another limit of FDG-PET/CT is its false-negatives rate encountered in case of small lesions (<1 cm, particularly <7 mm) [159], low tumor metabolic activity like bronchioloalveolar carcinoma [160], or hyperglycemia. Some authors have proposed dual-time point FDG-PET/CT imaging, using the change in SUV between early and delayed scans to help differentiate benign from malignant lesion. However, even if dual time point FDG-PET/CT appears to be more specific than single time point FDG-PET/CT (73% *vs.* 59% respectively), the results of a recent meta-analysis indicate that dual time point FDG-PET/CT and single time point FDG-PET/CT have similar accuracy in the differential diagnosis of pulmonary nodules (Area Under Curve—AUC): 0.8244 *vs*. 0.8220) [161]. Despite of the limits of this imaging modality, FDG-PET/CT is one of the current mainstays of SPN evaluation. The ACCP evidence-based clinical practice guidelines (2nd edition) for the evaluation of patients with pulmonary nodules [162] recommend FDG-PET/CT if an indeterminate nodule is larger than 8 mm in a patient with low to moderate risk of malignancy (risk based on age, smoking history, exposure, cancer history). If CT scan shows growth of nodule, or patient has high risk of malignancy, tissue diagnosis is recommended.

## 3. PET for Interim Tumor Response Assessment

The definitive proof of the therapy effectiveness is improvement in survival. However, imaging is generally used to assess therapeutic effects earlier. Surrogate endpoints for survival should provide more objective and hopefully correct answers about the efficacy of treatment: the time to tumor progression and progression-free survival, when the disease recurs or progresses. Because cancers typically grow before they cause death, dimensional parameters provide readouts of tumor growth often considerably before the patients die of tumor itself. These metrics have been shown in some, but not all, cancers to be predictive of survival [163]. Current response assessment is based primarily on changes in tumor size measured with CT or other traditional radiological imaging modalities. The limits of anatomical imaging technique in tumor response evaluation will be mentioned in the next section of this article (see PET in tumor response). 

The same limits of anatomical imaging are evident for tumor response monitoring during treatment: it appears intuitive that a reduction of tumor size after therapy indicates a better prognosis than does an unchanged or increasing tumor size. However, this assumption is not necessarily correct since the kinetics of tumor growth (and re-growth) after interim assessment could overtake the prognostic meaning of tumor size assessment in a given time point during treatment. Furthermore, tumor responses as assessed by radiological imaging modalities may be inaccurate because of errors in tumor measurements, errors in selection of measurable targets, and inter-observer variability of measurements [164]. For this reason, considerable efforts have been put in the last decade to redefine more reliable criteria. The progress in the knowledge of neoplastic cell metabolism prompted researchers the use of PET to assess the effect of cytostatic treatment on tumor cell metabolism objectively and quantitatively. First reports using planar FDG imaging for treatment monitoring were published more than 20 y ago [165], and subsequent studies in the early 1990s suggested that tumor response might be identified earlier through changes in the FDG signal than through changes in measured size [166,167]. Because of a high tumor chemosensitivity, early tumor response assessment by functional imaging with a FDG-PET/CT scan performed after few cycles of chemotherapy has shown to predict with a very high accuracy the final treatment outcome in lymphoma [168,169,170,171,172,173]. Two main patterns of FDG uptake, resulting from different metabolic changes within the tumor, are observed in PET scans performed during chemotherapy in DLBCL and in HL. The first, resulting from a ‘freezing’ of the image fading during the chemotherapy-induced FDG uptake decline constitutes the net result of a dynamic balance between cell kill and re-growth; the second is a consequence of an abrupt and persistent switch-off of FDG uptake by the tumor and accessory cells [174]. These differences, depending on the number of chemotherapy cycles before interim PET and on a peculiar tumor histologic architecture, could also explain the variable performance of PET in these lymphoma subtypes [175,176,177].

A series of studies published in the millennium turnaround has stressed the higher accuracy of FDG-PET as compared to CT for monitoring tumor response to therapy because of its ability in differentiating residual viable neoplastic tissue from treatment-induced necrosis and fibrosis. Specifically, FDG-PET has been shown to be able to identify patients with a good response to therapy despite the presence of residual masses on CT. In malignant lymphoma as well as in several solid tumors, patients with negative PET results after completion of therapy have been found to have a favorable prognosis, in spite of evidence, in CT, of considerable residual tumor tissue.

In solid tumors, preoperative chemotherapy, the so-called neo-adjuvant treatment, has emerged in the last few years as a therapeutic approach to ease complete tumor resection and to improve final treatment outcome, with a consistent increase in disease-free survival. In this setting, patients without a response on FDG-PET may thereby undergo tumor resection earlier and avoid the side effects of ineffective therapy. Indeed interim PET proved as a reliable and robust prognostic tool for treatment adaptation in a number of solid neoplasms [178,179]. 

FDG-PET/CT has been extensively used for the evaluation of cytoreductive therapies. Several guidelines have been developed to permit quantitative or at least semi-quantitative assessments of changes, notably, the National Cancer Institute [180] and the EORTC (European Organization for the Research and Treatment of Cancer) guidelines [181]. It is expected that guidelines for patient preparation and protocols for image acquisition, reconstruction, and analysis will be broadly similar for treatment monitoring during cytoreductive therapies and cytostatic agents. For instance, it is just essential to perform a baseline scan before cytostatic treatment start, in order to pick up even a subtle FDG uptake change occurring during treatment. Some changes in FDG uptake could depend on pharmacodynamics, whereas others are associated with reduced tumor cell viability (depending on cytostatic treatment). For example, imatinib mesylate decreases tumor FDG uptake within hours to days of the commencement of treatment, whereas endocrine therapies, such as tamoxifen, increases FDG uptake within the same time frame. In general, effects occurring from hours to days after the initiation of treatment reflect pharmacodynamics (e.g., a direct effect on glucose transporter expression or hexokinase activity). Effects occurring after approximately 2–3 week or after 1–3 cycles of treatment are more characteristic of reduced cell viability [182]. 

Finally, before entering in the topic of interim tumor response assessment in different hematological and solid tumors, the reader should be informed on a number of warnings that should be used in interpreting the existing literature data. First, a consistent heterogeneity is found among different studies reporting on sensitivity and specificity of FDG-PET/CT for treatment monitoring. This depends mainly on the fact that some reports allude to sensitivity and specificity in detecting *residual disease* while other articles have focused on the ability of interim PET in detecting a *tumor response* [164]. Second, different metrics have been used to report PET results: Visual assessment of semi-quantitative methods. The former was used in chemosensitive disorders like lymphoma, where a complete response is observed in most cases after first-line chemotherapy [173,174]. 

In solid tumors, however, more subtle changes in the intensity of FDG uptake reduction occur, which are not easy recognized of standardized with visual assessment only [70]. Semi-quantitative methods comprehend tumor-to normal tissue ratio and SUV. Both parameters are affected by a number of variables, the most important being the image acquisition time. All these parameters have been the object of a wide debate, because SUV assessment and in general patients scanning across different PET centers lacks reproducibility and data comparison is impossible in this setting [183,184,185]. 

Early treatment response assessment with FDG-PET has been used as a helpful tool in a number of solid tumors (See Table 2). 

**Table 2:** Indications of baseline and interim FDG-PET in principal solid tumor.

Tumor	Initial Staging	Interim Assessment
Breast	+(+)	++
Lung (NSCLC)	+(+)	++
Colorectal	−−	++
HNSCC	++	+
Esophagus	++	++

Legend of Table 2: ++: Generally useful; +: useful in selected cases; −: not useful.

### 3.1. Lymphoma 

**Figure 1:**
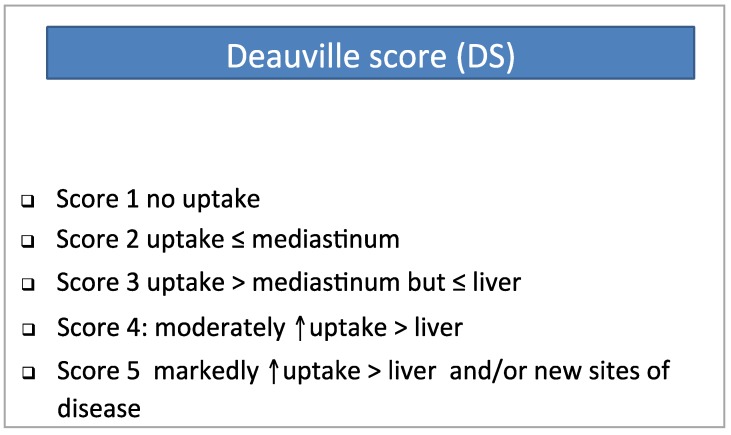
Deauville score (DS).

In HL, FDG-PET/CT enables an early evaluation of the metabolic changes occurring during the induction treatment as early as after the first [186], the second [170,171,172,187], and the third [188] cycle of chemotherapy (interim PET), with a predictive power on PFS even superseding the prognostic role of the International Prognostic Score (IPS) [189]. FDG-PET/CT yielded promising results as a surrogate of chemosensitivity for predicting tumor response also in DLBCL, albeit with a lower specificity [173,190,191,192], with an overall sensitivity and specificity of 43%–100% and 67%–100%, respectively [174] for both lymphoma subtype. This high performance of interim PET is mainly due to the high chemosensitivity of lymphoma. At this writing, it is unknown whether a PET-adapted a strategy based on early chemotherapy intensification in patients with a positive interim PET scan could improve the final treatment outcome in the whole cohort of HL patients; several trials, designed to answer to this relevant, clinical question are underway [193]. As mentioned above, the predicting role on treatment outcome is higher in HL as compared to DLBCL, and differences in tumor pathobiology such as the neoplastic architecture and different ratio of neoplastic clone to microenvironment cells could explain this different performance [176]. Other reasons could be the wide range of the number of chemotherapy cycles received before interim PET, the difference in patient selection criteria (retrospective/prospective, first-line or second-line treatment), the different criteria for scan reporting, and whether interim PET was done only for observational aims or in the context of a PET-adaptive strategy. Finally, interim-PET was performed in other lymphoma subtype, such as FL [194] and in peripheral T-cell lymphoma [195] with interesting, but still preliminary results. In April 2009 at a workshop on interim PET in lymphoma held in Deauville (France), simple and reproducible rules have been proposed for interim FDG-PET/CT visual interpretation [176,196], and these criteria have been recently retrospectively validated [197,198]. Briefly, the adopted rules include the following statements: (1) visual assessment is preferred, but SUV determination can be used in some cases; (2) interim PET interpretation should always be made by comparing the single foci of FDG uptake to those recorded in the baseline study; (3) the intensity of FDG uptake should be graded according to a five-point scale in which two reference organs, the mediastinal blood pool structures (MBPS) and liver, are used to define different grades of FDG uptake. Accordingly, the so-called five point scale has been proposed (Figure 1). The value of semi-quantitative FDG-PET/CT scan assessment was evaluated in the LYSA protocol LNH 2007-3B, in which interim FDG-PET/CT was performed after two (PET-2) and four (PET-4) cycles of chemotherapy and treatment adapted according to PET-4 results [173]. Optimal cutoff to predict treatment outcome was a Δ SUV_Max_ of PET-2 to PET-0 of 66% and of PET-4 to PET-0 of 70%. Outcomes did not differ significantly if PET-2 and PET-4 were reported by visual assessment. On the contrary, a Δ SUV_Max_ value of PET-2 to PET-0 ≥66% identified patients with a significantly worse prognosis with a 2-year PFS of 57% *vs.* 77%.

### 3.2. Breast Cancer 

Therapy response assessment in breast cancer patients is clinically relevant in cases with large and locally advanced tumors undergoing neo-adjuvant treatment with primary systemic chemotherapy. Schwarz-Dose *et al.* reported the SUV_Max_ reduction (ΔSUV_Max_) as semi-quantitative parameter to assess FDG-PET/CT predictive value with respect to pathological response [199]. A threshold of 45% reduction in SUV identified 11 of 15 responders, while the pathological non-responders were identified with a negative predictive value of 90% in the first cycle of chemotherapy. Martoni *et al.* and Keam *et al.* recently published similar results in patients undergoing neo-adjuvant chemotherapy [200,201]. Another unsettled issue is the optimal time point for interim FDG-PET/CT execution during neo-adjuvant chemotherapy treatment [1]. A number of studies reported a significantly higher SUV_Max_ reduction as early as after the 1st or 2nd chemotherapy course in patients with histological evidence as compared to chemo-resistant ones [202,203,204]. Smith *et al*. in a small cohort of 22 breast cancer patients found that all the responders had a SUV reduction ≥55% as compared to pre-treatment value [203]. Overall accuracy in predicting treatment outcome after the 1st and 2nd cycle were 88% and 91%, respectively. 

### 3.3. Colorectal Cancer 

Despite a curative intent, disease relapse often occurs in patients with stage III advanced colon cancer [205]. In this setting, chemotherapy seems to have no role, as disease could be eradicated by surgery or because of tumor chemo-resistance, in absence of predictors of treatment response.

Therefore a risk-adapted strategy with chemotherapy intensification for patients with a poor-prognosis disease while sparing toxicity for chemo-sensitive patients would be desirable and cost-effective. 

In advanced colorectal cancer, a correlation between treatment outcome and FDG-PET/CT metabolic response after 1 or 2 months of chemotherapy has been reported [206,207,208], but results were considered inconsistent, mostly due to methodological issues in PET reporting in multi-metastatic disease [208]. Interim assessment as early as after one course of therapy seems to be the more promising approach with a very high negative predictive value (NPV) for non-responding patients a good and a high predicting value on overall survival [209]. However, procedure for scanner harmonization and protocol standardization for patient scanning image acquisition and reconstruction are in progress. For this reason a prospective multicenter clinical trial aimed at assessing the preoperative chemosensitivity testing with FDG-PET/CT as predictor of treatment benefit in Adjuvant stage III colon cancer has been launched on behalf of the Belgian Group for Digestive Oncology (BDGO), the so-called PePiTA Trial [205]. The trial will be conducted with a strict Quality Assurance and Quality Control program for scanner harmonization with a traditional ^18^F NEMA phantom and imaging generation and reconstruction to qualify PET centers to take part to the study. All the data will be centralized in the Core Lab of the study, at the Nuclear Medicine Department of the Jules Bordet Institute in Brussels. 

### 3.4. Head and Neck Squamous Cell Carcinoma

The usefulness of interim FDG-PET/CT scanning in patients with HNSCC has been illustrated in a number of reports. Induction chemotherapy (ICT) has been used to select patients for organ preservation and determine subsequent treatments in patients with locally advanced HNSCC. Yoon *et al.* recently evaluated the efficacy of interim PET after ICT in HNSCC patients who achieved only partial response (PR) after ICT to predict clinical outcomes subsequent combined chemo-radiation [210]. A SUV_Max_ of 4.8 on interim FDG-PET/CT could predict final clinical CR after combined chemo-radiation (100% *vs.* 20%, *p* = 0.001), PFS (median, not reached *vs.* 8.5 months, *p* < 0.001), and OS (median, not reached *vs.* 12.0 months, *p* = 0.001) with a median follow-up of 20.3 months in surviving patients. The same held true for a 65% decrease in SUV_Max_ (ΔSUV_Max_) from baseline after ICT for final clinical CR (100% *vs.* 33.3%, *p* = 0.003), PFS (median, not reached *vs.* 8.9 months, *p* < 0.001) and OS (median, not reached *vs.* 24.4 months, *p* = 0.001) prediction. Ceulemans *et al.* prospectively compared the predictive ability of PET scan performed during radiotherapy to that performed 4 months after irradiation in a cohort of 40 HNSCC patients. The performance of interim was lower than end-of therapy FDG-PET/CT in terms of sensitivity (28.6% *vs.* 78.6%, *p* < 0.001), and negative predictive value (NPV) (31% *vs.* 60%, *p* < 0.001) [211]. 

### 3.5. Non-Small Cell Lung Cancer 

The number of patients presenting with NSCLC stage IV disease has increased over time [212]. This increase is most likely the result of better staging, because metastatic disease is identified long before it causes clinical symptoms [213]. Neo-adjuvant, preoperative chemotherapy for NSCLC has been extensively investigated, but its role in patient management remains controversial. Although responses to several courses of neo-adjuvant chemotherapy have been observed in up to 49% of the patients, no randomized trials have shown improvement in survival by preoperative chemotherapy and surgery as compared with surgery alone. FDG-PET/CT was reported to predict response to chemotherapy [214,215,216]. Hoekstra reported the results of a prospective multicenter study in which FDG-PET/CT was performed in 47 NSCLC patients before induction chemotherapy and after one and three cycles, and residual disease assessed in mediastinal lymph nodes [214]. Mediastinal lymph node status after induction chemotherapy by FDG-PET/CT predicted OS (*p* = 0.04) and PFS (*p* = 0.002). FDG-PET/CT was able to single out patients with different treatment outcome in the subset that showed treatment response according to CT. A positive FDG-PET/CT after one chemotherapy cycle predicted a poor PFS (*p* = 0.01). In a similar study, Decoster *et al.* prospectively assessed the predictive value of interim-FDG-PET/CT after 1 cycle of chemotherapy, compared to standard radiological assessment, according to the WHO recommendations. The concordance between the two imaging techniques was moderate (Spearman *r* = 0.62, *p* < 0.01). Surprisingly an early complete metabolic response did not improve patient overall survival [216].

More recently, several molecular-targeted agents such as the epidermal growth factor receptor tyrosine kinase inhibitors (EGFR-TKIs) erlotinib and gefitinib have emerged for treatment of NSCLC. Aukema *et al.* recently investigated the feasibility and the efficacy of early response monitoring with FDG-PET/CT during neo-adjuvant therapy with erlotinib in a small cohort of 23 NSCLC patients eligible for surgical resection [213]. Patients received preoperative erlotinib (150 mg) once daily for 3 weeks. FDG-PET/CT was performed before and at 1 week after erlotinib administration. Changes in tumor FDG uptake during treatment were prospectively assessed by SUV_Max_ measurement according to EORTC criteria [181]. According to these criteria, six patients (26%) had a partial response within 1 week, 16 patients (70%) had stable, and one patient (4%) had progressive disease. The median percentage of necrosis in the resection specimens of treated patients was 40%. In patients classified as “metabolic responders” (ΔSUV_Max_ ≥ 25%), the median percentage necrosis in the metabolic responder group was 70%, while the median percentage necrosis in metabolic non-responders was 40% (*p* = 0.09). In conclusion, early metabolic response corresponded to pathologic tumor regression in the resection specimen in most patients.

### 3.6. Esophageal Cancer 

Esophageal cancer is among the ten most common malignancies worldwide and is associated with a high mortality [217]. In patients with locally advanced esophageal cancer, preoperative chemotherapy or chemo-radiotherapy has been shown to improve outcome with respect to survival. Patients who respond to neo-adjuvant therapy have a significantly improved survival, compared with patients who do not respond to the therapy; in this setting FDG-PET/CT could be the ideal tool to assess chemosensitivity early during treatment, but shared interpretation criteria are still matter of debate. 

In one of the largest studies published so far on 211 consecutive patients who received neo-adjuvant chemotherapy followed by surgery, Miyata *et al*. reported the use of two semi-quantitative parameters for FDG-PET/CT scan interpretation: absolute post-treatment SUV_Max_ value (post-SUV_Max_) and ΔSUV_Max_ of interim from baseline FDG-PET/CT [218]. FDG-PET/CT was performed before and 2–3 weeks after completion of neo-adjuvant chemotherapy. The mean reduction of SUV_Max_ from baseline value (ΔSUV_Max_) was 49.4 (from 11.4 to 5.8). Δ SUV_Max_ and the SUV_Max_ value at the end of treatment (post-SUV_Max_) were able to predict pathologic response but not to distinguish partial from complete pathologic response. ΔSUV_Max_ ≥ 50% was predictive of a superior 5-year overall survival (56.5% *vs.* 39.6%, *p* = 0.01) as did a post SUV_Max_ < 3.5 (62.2% *vs.* 35.1%, *p* < 0.0001). 

Weber *et al.* monitored the response to treatment with FDG-PET/CT early in the course of therapy in 37 patients with locally advanced adenocarcinoma of the esophago-gastric junction [219]. FDG-PET/CT was carried out at baseline and 14 days after initiation of cisplatin-based polychemotherapy. For the quantitative assessment of therapy response, a circular ROI (diameter, 1.5 cm) was placed over the tumor in the slice with the maximum SUV in the baseline scan. In the second FDG-PET/CT scan, the ROI was placed at the same position as in the baseline study. The authors showed that FDG-PET/CT allowed prediction of pathological response by metabolic response assessment as early as 2 weeks after the chemotherapy onset. They established a cutoff value of more than 35% in baseline mean SUV that allowed the prediction of clinical response with a sensitivity and specificity of 93% and 95%, respectively.

On the basis of those findings FDG-PET/CT-response adapted clinical trials have been launched in single or multiple center settings. In the single-center MUNICON trial to prospectively evaluate the feasibility and potential effect on prognosis of administering FDG-PET/CT-response-guided chemotherapy 110 patients were evaluable for early metabolic response assessment after 2 weeks of induction chemotherapy [220]. 

Patients showing evidence of a metabolic response on FDG-PET/CT kept straight on with the original treatment for a maximum time of three months, while in case of no response patients underwent surgical resection two weeks later. 

A histopathologic response was achieved in 58% of the metabolic responders. After a median follow-up of 2.3 years, median overall survival was not reached in metabolic responders, whereas median overall survival was 25.8 months in non-responders (*p* = 0.015). Early assessment with FDG-PET/CT of the response to therapy in patients with locally advanced adenocarcinomas has shown promising results in single-center studies and should now be evaluated in randomized, prospective multicenter trials. Such trials are very important because they (1) could pave the way to a possible implementation of this strategy in clinical practice; (2) establish the minimal requirement for inter-scanner calibration and standardization of image acquisition and reconstruction in order to make semi-quantitative parameters such as SUV reproducible and reliable [221]. 

## 4. PET for Tumor Response 

### 4.1. Radiological Imaging for Tumor Response

The reduction in tumor size has been for decades the mainstay for monitoring the chemotherapy response in oncology. The World Health Organization (WHO) in the early eighties defined standard criteria for the assessment of tumor response [222]. The tumor bulk is defined as the sum of all nodal and extra-nodal lesions. The volume of nodal lesion should be measured by multiplying the 2 largest perpendicular diameters. The response to treatment is defined as a reduction ≥50% of the nodal and extranodal lesions. In case of complete disappearance of all the visible lesions the response is complete otherwise, the response is classified as a partial response. Twenty years later, the Response Evaluation Criteria in Solid Tumors (RECIST) for treatment response assessment [223], and subsequently, a revised version of the same criteria assessing a maximum of 5 tumor foci, *vs.* 10 in RECIST [224] was proposed, in order to simplify response assessment in clinical practice. 

RECIST criteria were developed moving from the concept that tumor shrinkage is a surrogate for assessing tumor response. As in the WHO proposal, anatomical, cross-sectional imaging obtained with CT or MRI was the underpinning of this proposal, with a slight difference, stating that only the longest nodal diameter measurement was enough for assessing tumor bulk and chemotherapy response. 

The issue of reproducibility of tumor size and tumor shrinkage measurement by using diameters of the lesions in trans-axial imaging techniques such as CT has been addressed by Moertel and Hanley in 1976 [225]. An experimental phantom was assembled filled with solid wood spheres embedded in a rubber matrix. Standard CT images were obtained and reproducibility of size measurement with rulers and calipers was checked among a panel of sixteen expert reviewers.

A disagreement on size measurement by 25% among reviewers on spheres with the identical diameter was present in 25% of reports and a disagreement of 50% in size in only 6.8% of cases. Although RECIST criteria have been used quite extensively in the past, some problems in tumor volume measurement were not the only pitfall in RECIST 1.0 and 1.1 versions: one issue is the choice of reducing intrinsically continuous phenomenon of tumor shrinkage and size reduction to a series of 4 levels of response (*i.e.*, complete response, partial response, stable disease, and progressive disease). Having reduced a continuous phenomenon in few, pre-defined categories of response could have led to a loss of important information [226,227]. 

### 4.2. The Problem of Residual Mass at the End of Treatment 

Another debated issue is the problem of residual mass at the end of treatment in several cancers and the inability of CT-based traditional radiological means to detect the persistence of tumor viable cells within a residual lesion harboring the tumor at baseline prompted the introduction of functional imaging for tumor response assessment in oncology.

This phenomenon was first described in lymphoma, where despite a good response to therapy, a residual mass can be demonstrated by radiological means in up to 80% of HL and in up to 40% of DLBCL patients after completion of treatment [228,229,230], even if only less then half of these masses will harbour residual disease [231]. Tumor masses often persist at the end of an adequate treatment for the presence of fibrotic tissue or extensive tumor necrosis. For the above reason the category of complete remission unconfirmed (RCU) was purposely proposed in the International Workshop criteria (IHP) for lymphoma response with the awareness that anatomic response criteria often underestimate the chemotherapeutic effect [232]. The significance of a residual mass at the end of treatment has been questioned also in a number of solid neoplasms. In HNSCC, Porceddu *et al.* [233] found the presence of a residual mass in 50/112 (45%) consecutive patients in CR who achieved a complete response at the primary site. The patients underwent CeCT and FDG-PET/CT for nodal response assessment at 12 weeks after the end of (chemo) radiotherapy. Forty-one of 50 patients with a residual mass at CT showed a negative FDG-PET/CT at 12 weeks after therapy and none of them relapsed after a median follow-up of 28 months after CR entry. In patients affected by gastro-intestinal stromal tumors (GIST), a metabolic response of the tumor, documented by a negative FDG-PET/CT scan preceded by weeks the anatomical response detected on CT. By contrast, in presence of an active metabolic tissue, as observed in absence of FDG-PET/CT response at the end of treatment, is consistent with primary chemo-resistance. A positive FDG-PET/CT observed during follow-up in a patient with a completely negative FDG-PET/CT at the end of treatment points toward a secondary chemo-resistance [234]. 

Similar findings of very good or complete response to treatment in presence of a limited tumor size-response assessed by RECIST criteria, have been reported in hepatomas treated by sorafenib [235,236]. In the SHARP trial, the efficacy of sorafenib, and inhibitor of vascular endothelial growth factor (VEGF) and of platelet-derived growth factor (PDGF) was assessed in 602 patients affected by Hepatocellular Carcinoma (HCC) randomly assigned to assume sorafenib or placebo. Partial response by RECIST criteria was observed in 2% and 1%, respectively. OS was slightly superior in the treated *vs.* placebo group (10.7 *vs.* 7.9 months, respectively; *p* = 0.001); however, the median time to radiologic progression in the sorafenib was significantly higher than in the placebo group (5.5 *vs.* 2.8, respectively; *p* < 0.001) [236]. In the same way in a number of hematological and non-hematological pediatric tumors the question whether a residual mass at the end of treatment is necessarily an harbinger of treatment failure could only be addressed only by functional imaging with FDG-PET/CT while traditional radiological imaging failed [237,238]. Thus, dimensional criteria proved to be no longer the more appropriate criteria in several neoplastic disorders. 

The concept that tumor bulk decreases over time during cytostatic treatment was proven in experimental animal tumor models. Moreover, a strong relationship between FDG uptake entity and cancer cell number has been reported in a substantial number of studies [239,240]. Therefore, a decline in FDG uptake during tumor shrinkage results from reduction of viable tumor cell number, while a sustained increase in tumor activity with increasing SUV values is seen upon tumor regrowth. Such a relationship, however, is not linear and seems lost for very small tumor burden where the entity of the residual tracer uptake by the tumor is beyond the detection power of FDG-PET/CT scan. In these settings, the definition of complete response of the tumor to an ablative cytostatic treatment does not lie on objective findings but is inductive [241,242]. In most neoplastic disorders, the tumor mass at diagnosis corresponds to a weight of 10–100 gr. or 10^10^–10^11^ cells. Cytostatic drugs induce cell death by first-order kinetics in a constant fraction of the neoplastic population; a given dose will kill a fixed fractional number and not a constant absolute number of cells, whatever the dimension of the tumor. Thus a single dose of a cytostatic drug able to induce 1 log of neoplastic cell loss in a tumor bulk equivalent to 10^11^ cells should repeated 11 times to completely dissolve the tumor [243,244]. 

The resolution ability of the commercially available scanners is able to detect residual mass with a diameter ranging between 0.4 to 1 cm, which translates in a tumor size corresponding to 0.1–0.5 to 1 gr., or 10^8^ to 10^9^ cells [245,246]. As a consequence, the range detection of a FDG-PET/CT in tumor staging and restaging, for tumors in apparent complete remission, is only two logarithms. In conclusion, a complete metabolic response at the end of treatment could be observed in tumors with a very broad mass range, corresponding to a difference in neoplastic total cell number as large as 7 logarithms [163]. Thus, in spite of its favorable prognostic meaning a negative FDG-PET/CT could be compatible with the presence of residual tumor cells. More in general, FDG-PET/CT negativization at the end of treatment or, in some cases, even during treatment as observed in different types of cancer, should be considered not yet a complete tumor eradication but rather a very good prognostic indicator of treatment response and, possibly, long-term disease control achieved by an efficacious antineoplastic treatment. 

Finally, three concepts should be well borne in mind: (1) several studies have shown that FDG-PET/CT is unsuitable to distinguish between minimal tumor burden *vs.* no tumor; (2) the scanners currently used are unable to detect microscopic residual tumor; (3) attempts to increase sensitivity of FDG-PET/CT by considering a residual FDG uptake an harbinger of residual tumor conflict with the presence of unspecific inflammation induced by chemo- or radiotherapy, thus compromising the specificity of this reading [163]. The same concepts could be extended to interim FDG-PET/CT during treatment: an negative interim FDG-PET/CT after few cycles of chemotherapy does not mean an early complete response (and disappearance) of the tumor at this time point, but rather an efficient fractional cell kill induced by the chemotherapy, leading, very likely, to a long-term tumor control by the chemotherapy itself. 

### 4.3. Functional Imaging for Tumor Response 

The relationship between FDG uptake and tumor response was evaluated in pioneer studies where several scans performed during treatment documented progressive FDG uptake shutoff in responding cancers [166]. Thus a steadily decreasing FDG uptake over time, in most cases anticipates a complete pathological response at the end of treatment. 

This tumor behavior, originally observed in breast cancer, is maintained across several other neoplasms such as lymphoma, lung cancer, HNSCC and esophageal cancers [163]. 

For all the above consideration, despite the important contribute of the traditional radiological means to document in a reproducible way the tumor bulk reduction induced by chemotherapy, the biologic predictive value of FDG-PET/CT appears to be greater in chemosensitive cancer such as lymphoma, lung cancer, mesothelioma, breast and esophageal cancer. For the above reasons, especially for the evident limitation of anatomical imaging for tumor response assessment, new criteria combining radiological and functional imaging have been proposed for treatment response assessment. 

The EORTC criteria for tumor response assessment with FDG-PET/CT were proposed in the turn of millennium, in 1999. Briefly, a complete resolution of FDG accumulation is an indication of complete metabolic response. Partial metabolic response is characterized by more than 25% reduction of FDG uptake, stable metabolic disease shows an increase in FDG uptake of less than 25% or a decrease of less than 15%, whereas an increase of more than 25 is attributed to progressive metabolic disease [181]. 

Ten years later, in an attempt to combine the anatomical and functional imaging results from RECIST and EORTC response criteria, new “mixed” criteria were proposed, the ‘‘PERCIST’’—Positron Emission Tomography Response Criteria In Solid Tumors [163]. The most relevant innovation of these new criteria is that cancer response is expressed quantitatively as a continuous variable instead of being defined with four separate categories. The chosen unit of measure for this quantitative assessment is SUV_Peak_, the SUV value referred to a ROI of a sphere of tissue with a diameter of approximately 1.2 cm (to produce a 1-cm^3^ volume spherical ROI). SUL (SUV corrected for lean body mass) is used instead SUV. Residual FDG uptake by the tumor is defined by the sum of SUL_Peak_ in up to five tumor lesions. Residual pathological lesions should be compared with the corresponding baseline tumors and each baseline (pre-treatment) tumor SUL_Peak_ value must be 1.5 × mean liver SUL + 2 SDs of mean SUL. Due to a quantitative FDG-PET/CT assessment, FDG-PET/CT sites should adopt a protocol for patient scanning consistent with the National Cancer Institute and The Netherlands multicenter trial group recommendations, as well as a documented procedure for quality assurance and quality control for the procedures of scanner calibration [180,247]. 

The SUL_Peak_ variations between pre- and post-treatment scans are used as metric for response assessment. Metabolic complete response is defined as the complete clearance of all sites of FDG concentration. A partial decline ≥ than 30% of SUV_Max_ and 0.8 unit decrease in SUL_Peak_ between the most intense pre- and post-treatment lesions identify a partial response. The lesions considered are not necessarily anatomically coincident. An increase ≥ than 30% SUV_Max_ and a 0.8 unit increase in SUL_Peak_ or the appearance of new lesions are the criteria for progressive disease. An adjunctive criterion for disease progression is an increase ≥75% of TLG. 

PERCIST criteria have been tested in limited reports in colorectal and in small cell lung cancer [248,249,250] and in bone metastases [251] with result similar or identical to EORTC classification. However, due to the complexity of the proposed quantitative assessment of residual tumor(s), and the logistic hurdles to be passed-by to put in place a thorough program of Quality assurance (QA) and quality control (QC) for scanner calibration in a multi-center settings, PERCIST has not been extensively used in clinical trial nor in the daily clinical practice.

In qualitative assessment of FDG-PET/CT scan performed at the end of treatment the intensity of FDG uptake in the residual sites still tracer-avid is compared to reference organs such as muscle, mediastinal blood pool, liver and brain. This interpretation key seems particularly attractive in neoplasms responsive to chemotherapy such as lymphoma, where a complete negativization of FDG-PET/CT scan is expected at the end of treatment in most cases. In this setting the IHP criteria have been proposed for end-of-treatment PET interpretation, combining the results of CeCT scan and FDG-PET/CT using both dimensional criteria with CT scan and dichotomized (positive or negative) FDG-PET/CT results [252,253]. These criteria, however, were validated only in a small cohort of 54 patients [254]. Moreover, dimensional criteria for lymph node size assessment were not fully reproducible, showing the least accuracy for diameters between 15 and 20 mm [255]. 

In 2009 during an international workshop of hemato-oncologists and nuclear medicine experts held in Deauville, simple and reproducible criteria for the interpretation of FDG-PET/CT performed interim during treatment by visual assessment were proposed for HL and DLBCL [196]. Here the residual FDG uptake assessed in the foci of persisting disease were compared to the FDG uptake in the so-called “reference” organs (mediastinal blood pool and liver), and scored along a five-point scale (5-PS) (See Figure 1 [196,256]).

Four years later, during the 12th International Congress on Malignant Lymphoma held in Lugano (Switzerland) in June 2013, a closed workshop attended by a group of haematologists and nuclear medicine experts was held with the aim of proposing and validating new response criteria for interim and end-of-treatment FDG-PET/CT in lymphoma, based on results of CeCT and FDG-PET/CT. Different criteria were proposed for FDG-avid and FDG-non avid lymphoma. For FDG-avid lymphoma (>90% of lymphoma subset) the 5-PS (See Figure 1) was proposed as interpretation key to quantify the entity of residual FDG uptake in FDG-PET/CT, along with dimensional criteria on CeCT. According to this proposal, patients with a score 1 to 3 were considered have complete metabolic response, patient with score 4–5 have partial response or progressive disease. Different from interim FDG-PET/CT, in which the 5-PS interpretation criteria were validated [197], in the case of FDG-PET/CT performed at the end of treatment these criteria were just a working proposal, awaiting confirmation in prospective studies. Furthermore, the choice to adopt the same cut-off value between score 3 and 4 to distinguish a negative from a positive FDG-PET/CT was somehow attenuated by the claim that “score 3 probably represents complete metabolic response (CMR), but patients may have different outcomes compared with score 1, 2, depending on clinical context and treatment regimen” [257]. For non FDG-avid lymphoma (SLL/CLL lymphoma, extra-nodal marginal zone lymphoma, lymphoplasmacytic lymphoma, mycosis fungoides, and other cutaneous lymphoma), only classical dimensional criteria, like in RECIST, were proposed. In this lymphoma subset the size of the residual tumour bulk should be assessed on CeCT, using the sum of the longest diameter of all the residual pathological lesions (See Table 3). 

**Table 3:** New Lugano Criteria.

Response Assessment at Interim	PET-CT Findings at Interim	Remission Assessment at End-of-Treatment	PET-CT Findings at End of Treatment
Complete Metabolic Response (CMR)	Score 1, 2 Score 3 also likely represents a good response at interim but an end-of-treatment scan is recommended for further evaluation.	Complete Metabolic Response (CMR)	Residual mass of any size and Score 1, 2. Score 3 should be interpreted according to the clinical context and pre-treatment prognosis but in many patients indicates a good prognosis/CMR with standard treatment. For trials where de-escalation strategies are being investigated, it may be preferable to consider score 3 as inadequate response to avoid under-treatment.
Partial metabolic response (PMR)	Score 4 or 5 & reduced uptake from baseline	Residual metabolic disease (RMD)	Score 4 or 5, with reduced uptake from baseline & residual mass of any size (but no new lesions)
No metabolic response or Progressive Metabolic Disease (NMR/PMD)	Score 5 & no significant decrease in uptake or new FDG avid foci consistent with lymphoma	No Metabolic Response or Progressive metabolic disease (NMR/PMD)	Score 4 or 5 & no significant change in uptake from baseline or new FDG avid foci consistent with lymphoma or increase in uptake in previous disease foci

### 4.4. Breast Cancer

Two different clinical situations should be considered: the locally advanced, operable and the metastatic breast cancer. There is increasing clinical evidence for metastatic breast cancer and other advanced-stage solid tumors that FDG-PET/CT is the most accurate imaging procedure for assessment of the response at the end of treatment when both CT information and tumor metabolic activity are considered. In operable breast cancer chemotherapy treatment is administered before surgery. In this setting, the neo-adjuvant chemotherapy, (1) allows for a considerable tumor reduction before surgery; (2) can improve breast conservation rate [258]; (3) yields a pathological restaging in all the cases; (4) it achieves similar results to those obtained with initial surgery followed by conventional adjuvant chemotherapy in terms of disease-free survival, overall survival, and loco-regional control [259]. Although approximately 70% of operable locally advanced patients demonstrate a clinical response after neo-adjuvant chemotherapy, either on physical examination or on anatomic imaging, only 3%–27% achieve a complete histopathologic response [260,261,262]. 

Histopathology is often used as the reference standard for assessing the response to primary chemotherapy in breast cancer and pathologic complete response (pCR) is defined as the disappearance of the neoplastic cell in the biopsy tissue. However, Honkoop *et al.* found no difference in survival between patients with scattered microscopic foci of residual tumor cells and patients who achieved a pCR [263]. Moreover, despite the evidence of persisting neoplastic tissue, pathology seems to have a suboptimal predictive power: between 13% and 25% of patients in pCR experience a systemic recurrence within 5 years from pCR entry [264,265,266]. Furthermore, a number of patients showing persistence of neoplastic tissue do remain disease free for longtime. In metastatic breast cancer the most widely accepted and used surrogate parameter is tumor bulk reduction, usually assessed by RECIST criteria. However, since several cycles of treatment are needed before a change in tumor size can be assessed by anatomic imaging, functional imaging either at the end but also during treatment could, at least in theory, perform better [1]. A recent meta-analysis of the diagnostic performance of FDG-PET/CT in metastatic breast cancer included 18 articles published from January 1995 to June 2004: the median sensitivity was 92.7%, and the median specificity was 81.6% [267]. Furthermore, comparisons with conventional imaging procedures used for staging (CT, US, and MRI) revealed that metabolic whole-body PET has a distinct advantage: the ability to assess tumor viability after previous treatments. A peculiar aspect is treatment efficacy evaluation of bone metastases because post-treatment healing is coupled with osteoblastic activity, ant the latter in turn, causes unspecific FGD uptake in the bone. Du *et al.* retrospectively analyzed the diagnostic contribute of FDG-PET/CT for bone metastases detection [268]. Briefly, 146 lesions were classified as osteolytic (77), osteoblastic (41), mixed (11) or negative (17) based on CT aspect. An increased FDG uptake (>80% of the cases in the first two abnormalities, >60% in the third subset) was recorded on FDG-PET/CT. Most of them (80%) as well as the CT-negative lesions showed a shutoff of FDG uptake upon treatment. Only 14 large lesions and nearly half (48%) of the osteoblastic lesions maintained a sustained FDG uptake. Some of the formerly negative lesions became osteoblastic on CT. In conclusion, a clear discrepancy between morphologic aspect on CT and presence of viable neoplastic tissue on FDG-PET/CT was noted. 

### 4.5. Colorectal Cancer 

The role of imaging in colorectal cancer management has emerged in the last few years in parallel with the impressive progress in long-term disease control achieved by the modern treatment combining surgical resection and chemo-radiation. FDG-PET/CT has an important role in: (1) cancer (re)-staging in case of locally recurring or metastatic disease; (2) in detection of the site of recurrence in case of unexplained increase of CEA; and (3), in the assessment of residual masses after treatment. Here a only a brief mention will be made on treatment response monitoring after local ablative therapy of liver metastases, and monitoring radiotherapy and multimodality treatment responses in primary rectal cancer, while the emerging role of FDG-PET/CT in the prediction and evaluation of responses to treatment, such as monitoring chemotherapy responses has been reviewed in a detailed way in the section of cancer treatment monitoring. 

Radical surgical resection is the preferred therapeutic option in advanced colorectal cancer with liver metastases. However, because of widespread metastatic disease dissemination in liver, complete resection with adequate tumor-free margins and adequate liver function reserve cannot be achieved in all patients. Selective loco-regional therapies have been proposed as alternative choice to radical surgery: cryosurgical ablation or cryotherapy (CSA) [269], radiofrequency ablation (RFA) [270], radioembolization using Yttrium^90^ (^90^Y) microspheres [271,272]. 

Different imaging techniques have been proposed for patient monitoring after loco-regional treatment of liver metastases [273,274,275,276,277]. FDG-PET/CT proved as a very accurate tool to discriminate unspecific inflammatory changes from persisting neoplastic uptake [273]. However problems arise in assessing treatment efficacy after radio-embolization with microspheres of ^90^Y. In this setting the preferred imaging technique is ^90^Y bremsstrahlung CT-integrated single-photon emission computed tomography (SPECT) [278]. One drawback of this technique as, in general, for scintigraphy images is the low spatial resolution, for lesions ≤1 centimeter [279,280,281]. Compared to SPECT/CT, FDG-PET/CT is a major step forward in metastatic liver disease detection after ^90^Y microsphere treatment, allowing a direct imaging of ^90^Y microspheres. Coincident ^18^F and ^90^Y images could be acquired due to a decay of ^90^Y to zirconium^90^, a β+ emitter [282,283,284,285]. 

The mainstay of therapy for colorectal cancer is radical surgery with radical resection of the mesial structures to prevent local and distant disease recurrence. However, despite the above radical surgical approach disease dissemination after surgery is still an ominous event. Locally advanced disease is the disease stage more frequently responsible for tumor spread and radiotherapy is often needed to improve disease control [286]. 

For locally advanced rectal cancer, neo-adjuvant chemoradiation has been proven successful [287,288]. Pathological examination of the resected tumor is the standard method to assess neo-adjuvant treatment outcome. Results are available only a few days after surgery and consequently cannot be used to guide individualized surgical procedure. However, first, accurate restaging to assess the success of pre-surgical neo-adjuvant treatment is mandatory for further treatment planning [289]. Two meta-analyses showed that CT, MRI and US are highly accurate in the staging of untreated tumors because of anatomic details to detect a peri-visceral or adjacent organ involvement [290,291]. The situation is completely opposed when these imaging modalities are intended to restage the tumor after chemoradiation, because very low accuracies ranging from only 30% to 60%. Chemoradiation is expected to induce fibrosis or scarring of neoplastic tissue, and therefore radiological imaging in unsuitable for tumor restaging. On the other hand, tissue inflammation reduces the specificity of FDG-PET/CT since FDG selective uptake occurs not only in neoplastic but also in inflammatory tissue [292,293]. In a head-to-head study comparing anatomical with functional imaging, FDG-PET/CT predicted therapy outcomes significantly better than endorectal ultrasound, CT, and MRI. In the study of Amthauer *et al.* [294], FDG-PET/CT had a sensitivity of 100% and a specificity of 86% when a minimum post-therapeutic SUV reduction of 36% was used to define a response. The positive and negative predictive values were 93% and 100%, respectively. Finally, Janssen *et al*. reported the results of a prospective study on FDG-PET/CT assessment of neo-adjuvant chemotherapy outcome in 51 patients with rectal cancer [295]: All patients underwent FDG-PET/CT imaging both before and 2 weeks after chemoradiation followed by local radical surgery. Tumor regression grade was calculated in resected samples according to Mandard criteria: grade 1–2 being considered pathological responders, grade 3–5 non-responders [296]. The best cutoff for value SUV_Max_ reduction (Δ SUV_Max_) in post treatment FDG-PET/CT scan to differentiate responders from non-responders was calculated by the ROC curve analysis. The most accurate cutoff value found in the training set for ΔSUV_Max_ was 48%. The same value was found in the validation set, with an overall sensitivity to predict treatment outcome in the training and validation set of 64%–83% and 93%–100%, respectively. 

### 4.6. Non-Small-Cell Lung Cancer

Mortality for NSCLC has decreased dramatically in the last few years [297]. Since tumor stage remains in a multivariate analysis the only prognostic factor significantly correlated with survival among more than 169 different prognostic factors [298], the largest efforts to reduce mortality from this kind of tumor are directed to obtain an early tumor diagnosis: the earlier the diagnosis the better is the prognosis. Moreover, with the advent of functional imaging with PET the detection of unsuspected distant metastases became apparent much earlier and more patients once addressed to a curative treatment with a single modality became candidates to a more aggressive treatment because of a relatively good performance status. Moreover, fewer patients are likely to undergo futile thoracotomy for early stage disease and more patients are identified as requiring aggressive loco-regional or systemic treatment. At the same time the panoply of therapeutic options for NSCLC has dramatically widened, including more aggressive surgical techniques and the use of neo-adjuvant radiotherapy and chemotherapy regimens before surgery [299]. 

In a prospective study aimed at assessing the role of combined treatment with radiotherapy and chemotherapy in a cohort of 73 medically or surgically non-resectable, non operable, stage Ia to IIIb NSCLC, FDG-PET was compared with CeCT by MacManus *et al.* in treatment response assessment after radiotherapy or chemoradiotherapy [300]. Treatment response categories were defined as CR (complete response), PR (partial response), NR (non-response), PD (progressive disease). Concordant results were found in only 40% of the patients. Patients entering CR by CT were fewer than those in CR by FDG-PET/CT (10 *vs.* 34) and conversely more patients achieved PR or ND in the CT as compared to PET group (27 and two *vs.* 37 and 11, respectively. PD results were similar in both groups (10 *vs.* 9). Six cases were not assessable in the CT group. In multivariate analysis only PET response turned out significantly associated with a better survival as compared to classical prognostic markers (*p* < 0.0001). FDG-PET/CT showed a high predictive value on treatment outcome when erlotinib, an epidermal growth factor receptor (EGFR) inhibitor, was administered before radical surgery as neo-adjuvant treatment in NSCLC: in 70% of the metabolic responders tumor necrosis had occurred, whereas necrosis was observed in only 40% of the non responders at 1 week after therapy [213]. Similar results were reported by Benz *et al.* [301] on FDG-PET/CT scan performed 2 weeks after the start of the neo-adjuvant erlotinib treatment, showing that an increasing metabolic activity in end of therapy PET was associated with a shorter time to progression (TTP) and a lower OS as compared to a longer TTP and OS for patients showing a stable of decreased FDG uptake (47 *vs.* 119 days and 87 *vs.* 828 days, respectively; *p* < 0.01 and <0.001, respectively.

### 4.7. Head and Neck Squamous Cell Carcinoma

Several reports aimed at assessing the predictive value on treatment outcome FDG-PET/CT after radiation or combined modality treatment in HNSCC have been published. Prestwich *et al.* retrospectively assessed the overall accuracy of FDG-PET/CT in response assessment in locally advanced HNSCC for primary tumors or nodal disease [302]. The sensitivity, specificity, positive predictive value and negative predictive value for primary tumor and nodal disease were 100%, 89%, 43%, 100%, and 100%, and 92%, 63%, and 100%, respectively. Due to this high performance the authors concluded the FDG-PET/CT performed in this setting could be decisional for HNSCC patients. In another study the role of FDG-PET/CT at the end of treatment of HNSCC was prospectively assessed: when FDG-PET/CT was performed 2 weeks after radiation therapy with a sensitivity and specificity of 86% and 85%, respectively in a small cohort of 32 patients [303]. For relapse detection at 4 months, the sensitivity was reported to be 92% before treatment and 2 weeks and 4 months after treatment. Porceddu *et al.* assessed the value of FDG-PET/CT to predict the presence of residual viable tumor tissue within CT-detected residual masses three month after chemo-radiotherapy in a cohort of 112 consecutive HNSCC patients [233]. Fifty patients had a residual mass detected on CT; in 41 of them the FDG-PET/CT was negative. After a minimal follow-up of more than two years, none of these 41 with a FDG-negative residual mass and none of the 21 PET-negative patients without a residual mass experienced treatment failure.

### 4.8. Epithelial Ovarian Cancer

Ovarian carcinoma is the first cause of death in gynecologic neoplastic disorders characterized by a widespread presentation, an aggressive course and a very dismal prognosis, with less then 44% of the patients surviving more than 5 years after diagnosis. Nearly half of the patients acquire complete remission after first-line treatment and the median PFS is about one year and a half [304]. Treatment failure is observed in 20%–30% of the cases due to early disease progression or no treatment response. A similar proportion of patients experience disease relapse within the first 6 months after treatment end [305]. In ovarian carcinoma as well as in several other neoplasms a prompt diagnosis of disease recurrence is recommended in order to start as soon as possible a new salvage treatment. In this perspective, several combinations of imaging techniques with serial monitoring, a number of biomarkers have been proposed for a prompt diagnosis of disease relapse. 

Significant controversy exists as to whether an earlier diagnosis of impending relapse with any of these modalities ultimately could improve patient survival [306,307,308,309]. FDG-PET/CT performs better than CeCT and MRI, particularly in the setting of suspected recurrence [310,311] and proved very useful in treatment decision to identify suitable patients for surgical resection from those in which chemotherapy is the only possible option [312,313]. In two small retrospective studies FDG-PET/CT was able to detect tumor relapse in presence of rising levels of CA 125 and equivocal CT findings or in case of symptoms and a negative CT, respectively [312,314]. In the first study FDG-PET/CT proved more accurate and precise than CT alone for localizing relapsing tumor with a sensitivity and specificity of 94.5% and 100%, respectively [311]. Furthermore, in the study of Bhosale *et al.* 31% of patients with no evidence of disease on CT had lesions present on FDG-PET/CT [314].

However, subsequent studies showed the inability of FDG-PET/CT to detect small volume disease <1 cm with FDG-PET/CT [315]; in those cases, CeCT scan should be planned whenever an increasing value of serum CA 125 is recorded or in presence of symptoms related to an impending relapse. FDG-PET/CT would be considered as a second-option imaging technique for patients candidates to second-line chemotherapy. 

## 5. MTV in Oncology 

Quantitative parameters for FDG-PET/CT interpretation proved as very robust prognostic parameters for treatment monitoring in oncology [163]. SUV is very easy to measure; it is readily available during PET interpretation and is an operator-independent variable. Its value has been considered to be strictly dependent from tissue glucose metabolism, provided that FDG-PET/CT are acquired in a standardized manner and proper scanner calibration procedures have been set up. Since the origin, different semi-quantitative and quantitative/kinetic analyses have been used to assess tumor metabolic response [316,317], but the methodology for determining total lesion glycolysis is still evolving; Different protocol for metabolic tumor volume assessment have been proposed with varied complexity of mathematic analysis, ranging from the more complex models of kinetic studies to methods based on tumor to background gradient of FDG uptake [316,317,318]. While full kinetic analysis required advanced sophisticated mathematical models and dynamic image acquisition, FDG-PET/CT allows a direct assessment of residual viable cell at a given time point during treatment. Moreover, a high tumor-to-background ratio encountered in most malignancies allows straightforward, automated and reproducible tumor delineation and determination of MTV. Interestingly, upon MTV multiplication by SUV_Mean_, TLG could be calculated. Automatic tumor delineation in FDG-PET/CT images is highly desirable for improved quantification, objective patient monitoring, and refinement of CT-based treatment planning in radiotherapy. However, the tumor segmentation task is challenging given the modest spatial resolution and the relatively high noise level in PET images. A large number of approaches have been proposed to segment tumors in PET images and the relative advantages or drawbacks discussed [7]. To date, there is no consensus on which method should be preferred for tumor segmentation, because of the difficulty in assessing tumor volumes *in vivo* [319]. Moreover, comparing the performance of these methods from the data published in the literature is almost impossible given the variety of situations in which evaluation studies have been conducted [7]. Finally, comparison of different segmentation methods on phantom-based studies could suffer from the impossibility of consider respiratory motion and heterogeneity of tumor FDG uptake. 

### 5.1. The Visual or Gradient Segmentation Method: The First One Applied and Still Widely Used

The sharp variation of activity measured across the external edge of the tumor (the so-called tumor to background ratio: T/B) is the underpinning of the methods this gradient to calculate the tumor volume. Other similar, but more sophisticated methods including a denoising or deblurring filter or based on T/B gradient estimation have been proposed [320]. The tumor segmentation MIM (Software Inc., Cleveland, OH, USA) vista based on tumor to background gradient described by Werner-Wasik *et al.* relies on calculation of spatial derivatives measured along six radii moving from a starting point in the centrum of the tumor lesion, identified by the operator [321]. The measurement of T/B gradient along these six radii provides the measures for tumor calculation. It has the advantage of being easily applicable from a technical point of view but is affected by a significant inter-observer variability [322] and it is time consuming.

### 5.2. Fixed-Percentage Threshold Segmentation Method

A totally different approach is used to measure the tumor volume based on a fixed threshold of SUV_Max_ method. In brief, all the voxel measured within a pre defined sphere inside a given tumor mass exceeding a pre-defined percentage of SUV_Max_ value are considered to measure the tumor volume of this mass. Cross sectional circles in three orthogonal plans are considered to cover all the mass volume [321]. Due to biological and physical factors there are no “normal” values for SUVs to be similarly used in every case. It has been shown that this method often fails, e.g., when the physiological background activity lies above the fixed threshold [323].

### 5.3. Maximal Intensity Threshold 

New methods for tumor segmentation have been proposed such as the maximal intensity threshold, based on calculation of all the voxel exceeding an absolute SUV_Max_ value. These methods were deemed more accurate for tumor identification and volume measurement compared to fixed-threshold ones [323,324]. However, criticism against these conclusions arose from the phantom studies, showing a superiority of gradient segmentation model for tumor quantification [321]. The error was low (<5%) for tumor lesions with a diameter > than 2 cm. whatever the segmentation method used, while a significantly lower error value (8.2%) was found adopting a T/B gradient method compared to a 45% maximal intensity method for lesions with a diameter ≤2 cm. Moreover, despite background noise measurement depends on acquisition time, the later does not seem to affect the results of T/B gradient methods for MTV calculation [321].

### 5.4. Adaptive Threshold Segmentation Method

According to this method initial tumor volume estimate is determined on CT images. Approximate source to background (S/B) are then obtained for the corresponding tumor lesion on PET images. From the CT estimate of the lesion size and the PET estimate of the S/B ratio, the appropriate optimum threshold is chosen. The threshold is applied to PET images to obtain lesion activity and a final estimate of the lesion volume [325].

### 5.5. Methods of Tumor Contour

To calculate MTV and TLG a preliminary tumor mapping with a manual contouring of all the tumor lesions by nuclear medicine physicians has been proposed. However, this procedure proved cumbersome when several lesions coalesce in a single bulky tumor mass, and time-consuming for the analytic measurement of SUV_Max_ in every tumor lesion in advanced, stage disseminated metastatic disease. With the semi-automatic method only two manual interventions are needed: the first consists in the identification of spatial orthogonal axes of the tumor mass. Then the dedicated software automatically calculated the tumor volume along these three axes as in MIM vista method (see above), and finally the operator manually adjusts the shape of the contour to fit the anatomical edge of the tumor mass [9].

### 5.6. Other Methods

Many new methods are still threshold-based, but either automate the choice of SUV threshold specific to an image [326,327] or apply thresholds to a combination (e.g., ratio) of SUV and an image-specific background value [328,329]. More segmentation algorithms are entering PET oncology from the field of computer vision [330] including the use of image gradients [320], deformable contour models [331,332], mutual information in hybrid images [333] and histogram mixture models for heterogeneous regions [334]. The current staging methods in oncology are based on the appraisal of size, number and location of the neoplastic lesions at baseline but they do not provide accurate measurement of tumor volume. The latter, in fact, is only loosely correlated to its size; as an example, patients presenting with micro-metastases in lymph nodes may show the same N value in TNM staging than patients with bulky nodal lesions [335]. Data available in patients affected by HNSCC, lung carcinoma, esophageal carcinoma and gynecological malignancies suggest that MTV and to a lesser extent TLG have the potential to become valuable in the imaging of human solid tumors as prognostic biomarkers for short- to intermediate-term survival outcomes, adding value to clinical staging, for assessment of response to treatment with neo-adjuvant and concurrent chemotherapy, and for treatment optimization [336]. Based on early treatment response assessment using changes in metabolic tumor volume over time, it might be possible to select patients who require a more aggressive treatment to improve their outcome.

TNM staging system is currently the most widely applied prognostic system for patients with HNSCC, but it proved suboptimal for identification of patients at high risk of recurrence [337]. Chung *et al.* studied 43 clinically node-negative patients with oral HNSCC who had all undergone primary tumor resection and elective neck dissection in addition to a pretreatment FDG-PET/CT examination [338]. In order to correlate the prognostic impact of imaging results and surgical staging the following parameters were evaluated: age, gender, clinical T stage, SUV_Max_, MTV and depth of invasion, lympho-vascular invasion, pathological tumor volume and histological differentiation. In multivariate analysis, MTV proved the only relevant prognostic factor in predicting tumor spread (hazard ratio 54.66, CI 1.05–2842.86); patients with an MTV of >6 mL had significantly higher numbers of occult metastases. Dibble *et al.* [339] reported the value of MTV and TLG in a cohort of 45 patients with histologically proven oral or oro-pharyngeal HNSCC staged at baseline with FDG-PET/CT. The only parameters significantly associated with survival and treatment outcome were MTV and TLG. The median cut-off point of 7.7 mL for primary tumor MTV was the best predictive value on receiver operator characteristics (ROC) analysis. MTV and TLG in primary tumor and of lymph nodes were prospectively assessed by Chan *et al.* [340] in a cohort of 196 stage III/IVb patients affected by HNSCC of the nasopharynx, Combination of a TLG >330 mL and stage IV were predictive of local recurrence, while high SUV_Max_ of lymph nodes and stage IV were predictive of distant disease spread. 

In lung cancer, the FDG uptake intensity at baseline and after neo-adjuvant chemotherapy proved a powerful predictor of survival, and SUV_Max_ overtook TNM staging in predicting chemotherapy response [336]. Therefore the prognostic value of SUV_Max_ of the primary tumor in FDG-PET/CT studies for risk stratification in NSCLC patients has been increasingly used [341]. Lee *et al.* identified 19 patients with lung cancer (18 of them with NSCLC) staged and re-staged by FDG-PET/CT scans before and after therapy, and at the time of progression in 15 out of 19 experiencing tumor relapse [342]. Metabolically active tumor regions were segmented on pre- and post-treatment FDG-PET/CT scans semi automatically using custom software. Median MTV was 27 mL and, in multivariate analysis, an increase in MTV ≥25 mL in end of treatment was associated with increased hazard of progression and of death (5.4-fold and 7.6-fold), statistically significant (*p* = 0.0014 and *p* = 0.001) after controlling for stage, treatment intent, age, Karnofsky performance status, and weight loss. In a comprehensive large review on 11 (10 retrospective, one prospective) studies for a total number of 1,204 patients suffering from lung cancer (955 with NSCLL and 249 SCLL) both in limited and extended disease, Van de Wiele *et al.* [336] found that MTV in nearly all studies and in lower proportion, TLG were predictive of disease free survival overall survival or macroscopic/microscopic disease spread.

Among patients affected by esophageal carcinoma most present with widespread disease and, in spite of improvements in surgical and neo-adjuvant therapies, die for their disease within 1 year from diagnosis [343,344]. Resectable disease and TNM stage at the time of diagnosis are by far the most important prognostic factors. Additional factors are tumor length, the number and the proportion of involved lymph nodes. Tumor resection is possible in stage I-III, but in stage III, due to the presence of a locally advanced disease tumor control with surgery alone is poor, and neo-adjuvant chemotherapy seems useful in prolonging time to progression. 

Interim assessment of clinical response with FDG-PET/CT during neo-adjuvant treatment is required to permit early treatment adaptation [345,346]. To detect pathological responders from non-responders before surgical resection of locally advanced esophageal carcinoma, Jayachandran *et al.* calculated the predictive value of 0.5. In their series of 37 patients, both MTV2.5 and TLG2.5 (MTV and TLG defined using a fixed SUV threshold of 2.5) were predictive of response and survival, whereas SUV_Max_ was not [347]. It was suggested that FDG-PET/CT scan may potentially guide (surgical) therapy after chemo-radiotherapy. MTV assessment with FDG-PET/CT has been used to determine tumor length and locally advanced tumor bulk [348,349]. MTV2.5 (was also used in a study by Roedl *et al.* for metabolic tumor length determination on pre- and post-treatment scans after neo-adjuvant chemo-radiotherapy in a series of 47 patients, to check whether a tumor length decrease ≥33% was predictive of histopathological response [350]. Using this threshold, a sensitivity and specificity for predicting pathological response of 91% and 92%, respectively, were obtained, *versus* 86% and 61% using a threshold ≥43% SUV_Max_ decrease from baseline values. 

Between 20% and 40% of patients with cervix carcinoma of the uterus, treated with concurrent chemoradiation, will have recurrent or persistent disease [351]. As in other solid cancers the early identification of high-risk patients for treatment failure could pave the way to an intensified treatment for this patient subset. Kidd *et al.* have also shown in a series of 25 patients with stage Ib1–IVa cervical cancer scheduled to undergo chemoradiation that higher pretreatment MTV40% values (≥55.4 cm^3^) are significantly associated with poor treatment response in multivariate analysis [352]. 

In conclusion, despite the heterogeneity in the clinical behavior and aggressiveness of the neoplastic disorders, the prognostic meaning of MTV and TLG persist across different types of solid cancer and seems more evident in locally-advanced tumors, where the risk of disease recurrence after surgery is highest and the benefits of neo-adjuvant chemotherapy or chemoradiation more evident. Since TNM stage is often used to decide the indication of neo-adjuvant chemotherapy, MTV showed higher predictive value and superseded anatomical tools such as TNM staging to guide preoperative treatment. 

## 6. FDG-PET and Multiple Myeloma 

Multiple Myeloma (MM) is a hematologic neoplasm characterized by bone marrow invasion of a neoplastic clone of plasma cells, presence of bone lytic lesions and extra-medullary organ invasion in later stages of the disease [353]. The disease course is heterogeneous with a patient survival ranging from a few months to more than a decade [354]. In this perspective a still unfilled need exists for reliable prognostic tool identification in order to predict the individual disease outcome in a single-patient basis. As in other solid and hematologic cancers one of the most important prognostic factors is tumor dissemination (or stage) at baseline; the most frequently involved organ is bone and bone marrow but, due to the heterogeneity of the pattern of bone and bone marrow invasion by disease standardization and grading of tumor spread in this organ has been difficult [355]. 

The MM staging system was introduced in 1975 by Durie and Salmon to allow patient stratification with different tumor bulk by calculating the measurable theoretical myeloma cell mass [356]. However, the emergence in the last decade of new imaging techniques, as MRI and FDG-PET/CT have provided new insights on tumor spread at disease onset and gradually revolutionized staging system developing a more integrating clinical and imaging approach, the Durie/Salmon PLUS myeloma staging [357]. Sensitivity of FDG-PET/CT proved very high for detection of extra-skeletal lesions, while MRI outperforms FDG-PET/CT for detecting bone and bone marrow invasion by disease, especially for spine and pelvic localizations [358].

FDG-PET/CT proved a powerful prognosticator in MM: MTV measurement at baseline, and longitudinal studies aimed at assessing the role of minimal residual disease detection by FDG-PET/CT are the most innovative aspect of functional imaging in this disease. 

Durie and Salmon staging [356] and later on the International Staging System [355] have been proposed for tumor spread assessment, laying on a wide array of biochemical markers and conventional radiographic imaging for bone lytic lesion detection. However, a definite cutoff for serum albumin and beta2-microglobulin still lacks due to a frequently coexisting impaired renal function; furthermore, conventional radiography can significantly underestimate lytic lesions because up to 30% of the trabecular bone could be missed in standard radiographs. In this setting, a number of different diseases showing a wide range of tumor bulk and could be included in the same category of stage III MM. 

SUV_MAX_ measurement and MTV calculation are able to stratify stage III MM patients in different subsets according to tumor bulk, characterized by different prognosis and treatment outcome. These new insights on MM tumor burden originate from the pioneer retrospective study by Fonti *et al.* on the prognostic value of MTV assessed at baseline by FDG-PET/CT in a cohort of 47 stage IIIA MM patients treated with bortezomid and lenalidomide or thalidomide and autologous stem cell transplantation in half of the patients [359]. ROI for MTV calculations were defined as focal lesions detected on CT scan showing a SUV_Max_ ≥ 2.5 on PET. MTV was calculated using an automated contouring program based on a fixed threshold value of 40% of SUV_Max_. At follow-up, patients who developed progressive disease or dying for myeloma showed a significantly higher MTV (74.7 ± 19.3 *vs.* 29.8 ± 5.1 mL, *p* = 0.009 and 123.2 ± 29.8 ± 4.2, *p* = 0.0001). PFS was significantly prolonged in patients with MTV < 42.2 mL as compared with patients with MTV ≥ 42.2 mL (*p* = 0.0465) and OS was significantly better in patients with MTV <77.6 mL as compared had MTV ≥77.6 mL. In conclusion MTV seems to be closely correlated to plasma cell mass and may be a valid clinical help to correlate with patients outcome. 

Zamagni *et al.* analyzed the prognostic relevance of FDG-PET/CT on treatment outcome in a longitudinal prospective study [358]. In a cohort of 192 MM patients consecutively enrolled, FDG-PET/CT was performed at diagnosis, after induction therapy with thalidomide and dexametasone (TD), after double autologous stem cell transplantation (ASCT) and later during follow-up. Presence at baseline of at least three focal lesions (44% of cases), a SUV_Max_ > 4.2 (46%), and extra-medullary disease (EMD; 6%) adversely affected 4-year estimates of progression-free survival (PFS; >3 FLs: 50%; SUV > 4.2: 43%; presence of EMD: 28%). SUV_Max_ > 4.2 and EMD were also correlated with shorter overall survival (OS; 4-year rates: 77% and 66%, respectively). Patients with a negative baseline FDG-PET/CT were 24% and positive 76%. A diffuse bone marrow FDG uptake was observed in 17% while 59% had focal lesions with an SUV_Max_ > 4.2 in 46% and SUV < or equal 4.2 in 54%. This threshold of 4.2 in SUV could distinguish a 4-year PFS of 4.2% and 66%, respectively (*p* = 0.003) and 4-year OS of 76% and 92%, respectively (*p* = 0.02). After induction FDG-PET/CT-positive patients were 63%, the same SUV_Max_ threshold lower of higher than 4.2 was able to distinguish two distinct patient cohorts with a a 4-year PFS of 44% and 69%, respectively (*p* = 0.007) and a 4-year OS of 75% and 88%, respectively (*p* = 0.09). After ASCT 4-year PFS and OS for FDG-PET/CT-negative patients were 47% and 79%, respectively, compared with values of 32% (*p* = 0.02) and 66% (*p* = 0.02), respectively, for FDG-PET/CT-positive patients. 

In conclusion, with the limitations intrinsic to a quantitative analysis of FDG-PET/CT data in absence of information on scanner calibrations, image acquisition and reconstruction, and fasting glucose levels of the scanned patients, and of a retrospective nature of the study, this preliminary report seems to stress the relevant prognostic role of FDG-PET/CT throughout the during the natural history of the disease and could pave the way to prospective studies aimed at assessing a patient-adapted strategy for MM treatment. 

## 7. FDG-PET/CT in Surveillance of Cancer 

At this time, FDG-PET/CT is not routinely recommended in the surveillance of most cancers [360,361]. Actually, there is insufficient evidence to define the clinical impact of FDG-PET/CT in this indication [362]. This is the case for lymphoma, for which several studies observed a low PPV of FDG-PET/CT, with a high rate of false-positive results [363]. However, some data suggest that FDG-PET/CT may be interesting in surveillance and follow-up; this is the case of a prospective study of 91 HNSCC patients, which observed a high effectiveness of FGD-PET/CT in the assessment of the disease recurrence (sensitivity 100%, specificity 85%, PPV 77%, NPV 100%). In this study, FDG-ET/CT examination was performed 11.6 ± 4.4 months after the end of the treatment. In contrast, a recent meta-analysis observed more various rates of sensitivity and PPV (75%–100% and 50%–90%, respectively) for the surveillance of head and neck cancer [362]. For colorectal cancer, a randomized controlled trial of 130 patients reported that recurrences were detected after a shorter time (12.1 *vs.* 15.4 months; *p* = 0.01) in the PET group compared to the conventional group, and recurrences were also more frequently (10 *vs.* two patients) cured by surgery in the PET group [364]. The authors concluded that FDG-PET/CT may permit the earlier detection of recurrence of colorectal cancer, but once again, more data are warranted to confirm that. The potential risks of using FDG-PET/CT for surveillance are overtreatment caused by false-positives, and unnecessary radiation exposure. The lack of a common definition of surveillance (the minimal time since last treatment, the absence of clinical or other diagnostic suspicion of recurrence) is also an unsolved issue at this time [362]. In the case of surveillance of melanoma, the data are clearer [129], and FDG-PET/CT is a promising tool in this field. Finally, we should remember that FDG-PET/CT is a useful imaging modality in case of elevation of serum tumor markers, in particular in ovarian cancer [97].

## 8. New PET Technologies and Tracers

FDG is the most frequently used PET radiopharmaceutical, but other PET tracers are progressively developed and used, not only in literature publications, but also in clinical practice [365]. We will expose some example to illustrate this topic.

The main application of ^18^F-fluorocholine (FCH) and ^11^C-fluorocholine, some phospholipid cell membrane metabolism markers, is prostate cancer. FDG is also often suboptimal in prostate cancer, because of low tracer avidity, and FCH-PET/CT is a promising alternative molecular imaging in that field [367]. Its main indication is prostate-specific antigen (PSA) elevation, which has been demonstrated as a useful issue in several publications (pooled sensitivity of 85.6% and pooled specificity of 92.6% in the meta-analysis of Evangelista *et al.*) [367,368]. Due to the strong relationship between PSA kinetics and detection rate of FCH-PET/CT, it should be taken into account in the selection of prostate cancer patients who should undergo FCH-PET/CT for restaging [369]. A recent meta-analysis observed that FCH-PET/CT led to a change in treatment in 381 (41%) of 938 patients who performed FCH-PET/CT during staging and restaging for biochemical recurrence [368]. However, in staging of patients with proven but untreated, high-risk prostate cancer patients, there is limited but promising evidence warranting further studies; in this setting, Choline-PET/CT has a pooled sensitivity and specificity of 84% (95% confidence interval: 68%–93%) and 79% (95% CI, 53%–93%), respectively [370], and provides low sensitivity in the detection of lymph node metastases prior to surgery (pooled sensitivity 49.2%) [371]. 

For brain imaging, FDG uptake correlates with tumor grade, cell density, biological aggressiveness and survival in patients with primary or recurrent gliomas [372]. However, this approach can be limited due to the high physiological cerebral activity. Dual time point imaging which takes advantage of the slower dephosphorylation of tumoral FDG *vs.* normal brain improves tumor to normal brain contrast [373,374]. Fluorinated amino acids, 18F-fluoroethyltyrosine (FET) and 18F-Fluorodihydroxyphenylalanine (^18^F-DOPA) are an interesting alternative for brain tumor imaging due to the specific over-expression of amino-acids transporters in brain tumors unlike normal brain tissue. Both of them show comparable results to 11C-methionine PET and are able to help diagnosing tumor recurrence *versus* radiation necrosis, guiding stereotactic biopsy and treatment evaluation [375,376,377]. They are also useful in brain metastases evaluation [378]. Relative differences are still debated especially concerning low-grade gliomas evaluations [379].

^18^F-DOPA, the immediate precursor of dopamine, has also high diagnostic performances in adrenal and extra-adrenal paragangliomas [380] or in the detection of recurrent medullary thyroid carcinoma, and may be an interesting alternative imaging modality in neuroendocrine tumors [381].

Other tracers like ^18^F-fluorothymidine (FLT), a thymidine analogs, explore the cell proliferation and can be used to detect tumors in many areas of the body. We have to take into account that background uptake of FLT is high in the liver, marrow, and renal system, limiting use in these organs; actually, its most promising use is in monitoring tumor treatment response [382,383].

More specific tracers may also be used, like ^18^F-fluoride, an osseous marker which has higher image quality than SPECT-CT bone scintigraphy equivalent [365]. According to the SNM Practice Guideline for Sodium ^18^F-Fluoride PET/CT Bone Scans 1.0, PET/CT ^18^F-fluoride bone scans may be used to identify skeletal metastases, including localization and determination of the extent of disease [384].

Other specific radiopharmaceuticals exist, like ^124^I-Iodine for PET/CT imaging of thyroid cancer, ^18^F-fluoroestradiol for imaging of estrogen receptors in breast cancer, or ^68^Ga-labelled somatostatin analogue [365]. 

The main application of ^68^Ga generator is the exploration of somatostatin receptors. ^68^Ga-labelled somatostatin analogue PET/CT is useful for the staging of neuroendocrine tumors (gastro-entero-pancreatic or bronchial neuroendocrine tumors mainly), and seems to be superior compared to ^111^In-DTPA-octreotide SPECT-CT [385,386]. This radiopharmaceutical may provide additional diagnostic information in a high proportion of patients with consequent high management impact. ^68^Ga-labelled somatostatin analogue PET/CT could replace ^111^In-DTPA-octreotide scintigraphy at centers where it is available given its superior accuracy, faster acquisition and lower radiation exposure. Moreover, it offers the possibility to noninvasively evaluate neuroendocrine tumor cells for the presence of somatostatin receptor expression, with direct therapeutic implications [387].

In addition to new radiopharmaceuticals, new PET technologies, mainly FDG-PET/MRI, may offer new opportunities. Hybrid FDG-PET/MRI may be particularly interesting because of its superior resolution and soft tissue contrast (e.g., tumors in the brain, the head-and-neck region, or the pelvis), and its lack of ionizing radiation exposure [388]. Recent studies show the effectiveness of whole-body FDG-PET/MRI imaging in oncology, observing that FDG-PET/MRI performed comparatively to FDG-PET/CT in lesion detection and quantitative measurements [389,390]. Several potential applications are described in the literature: the evaluation of bone metastases from prostate cancer with simultaneous FCH-PET/MRI [391], the follow-up of head and neck cancer patients [392], the initial staging of pediatric lymphoma as a radiation-free alternative to FDG-PET/CT [393], or the lung cancer M-staging especially for brain and liver metastases [394].

## 9. Conclusions

During the last 20 years the continuous technological progress has revolutionized the role of PET scan in Oncology. Moving from its original role for tumor staging and restaging PET/CT has become a seminal tool for tumor prognostication: as a new metrics for tumor volume and spread measurement at baseline and as a compass for treatment tailoring both in lymphoma and solid tumors like esophageal carcinoma, inoperable non-small cell lung carcinoma and metastatic breast cancer. Challenges for its use with nuclear magnetic resonance in a single-shot scan, and as a unit of measure for tumor burden quantification are opening new frontiers for the medical imaging research.

## Abbreviations

FDG: 18-Fluorodeoxyglucose
PET: Positron Emission Tomography
CT: Computed Tomography
FDG-PET/CT: Combined Positron Emission Tomography and Computed Tomography
SPECT: Single-photon Emission Computed Tomography
MRI: Magnetic Resonance Imaging
US: Ultrasound
EUS: Endoscopy Ultrasound
BS: Bone Scintigraphy
SUV: Standardized Uptake Value
SUV_Max_: Maximal SUV Value
SUV_Mean_: Median SUV Value
SUV_Peak_: SUV value in a1-cm^3^ volume spherical ROI
ΔSUV: reduction in SUV_Max_
ROI: Region Of Interest
MTV: Metabolic Tumor Volume
TLG: Total Lesion Glycolysis
CeCT: Contrast-Enhanced Computed Tomography
BMB: Bone Marrow Biopsy
B/BM: Bone and Bone Marrow
PPV: Positive Predictive Value
NPV: Negative Predictive Value
AUC: Area Under Curve
ROC: Receiver Operating Charateristic
